# Shared Reality Can Reduce Stressor Reactivity

**DOI:** 10.3389/fpsyg.2022.853750

**Published:** 2022-04-27

**Authors:** Megan R. Goldring, Federica Pinelli, Niall Bolger, E. Tory Higgins

**Affiliations:** Department of Psychology, Columbia University, New York, NY, United States

**Keywords:** stressor reactivity, shared reality theory, tend and befriend theory, psychophysiology, daily diary

## Abstract

When a person faces a stressor alongside someone else, do they get more or less stressed when the other person agrees that the situation is stressful? While an equally stressed partner could plausibly amplify stress by making the situation seem more real and worthy of distress, we find that social validation during co-experienced stressors reduces reactivity. Specifically, the psychological experience of shared reality calms some people down. In Study 1, 70 undergraduate females who jointly faced a stressful event with someone else reported feeling less anxious when the other person felt the same way about the stressor, relative to when the other person appraised the situation in the opposite way or provided no indication of their appraisal. These findings were reflected in participants’ physiological reactivity, especially in the parasympathetic nervous system. In Study 2, we generalize these findings to co-experienced stressors in the daily lives of 102 heteronormative romantic couples in the New York City area. In line with tend-and-befriend theory, we found that shared reality during co-experienced stressors reduced anxiety for almost all females (99% of the sample) and for a minority of males (42% of the sample). Together, these findings unify major theories in health and social psychology by implying that shared reality reduces stressor reactivity, and that this effect is partially moderated by sex.

## Introduction

Around 60% of daily life stressors are faced alongside another person (Almeida et al., 2002). For example, siblings jointly cope with parent’s Alzheimer’s diagnoses, co-workers approach deadlines together, and university students mutually prepare for finals. However, the bulk of existing research on the social psychology of stress focuses on events that implicate only one person, while another person supports them (for a review, see Taylor, 2011), regulates their mood (Diamond and Aspinwall, 2003; Zaki and Williams, 2013), shares in their emotions with them after the fact (for a review, see Rimé, 2009; see also Steel et al., 2015), empathizes with them (for a review, see Cuff et al., 2016), or is merely present (Allen et al., 1991; Felnhofer et al., 2019; Qi et al., 2020). Although psychologists have long known the social nature of stress (Schachter, 1959), research on co-experienced stressors remains understudied.

Critical questions therefore remain. When a person faces a stressor alongside someone else, do they get more or less stressed when the other person agrees that the situation is stressful? What psychological process explains the costs or benefits of such agreement? And given sex differences in the tendency to *want* to be together while stressed (Schachter, 1959; Taylor et al., 2000; Taylor, 2006; Taylor and Master, 2011), do males and females respond differently to social validation during co-experienced stressors? In one psychophysiological experiment and one dyadic daily diary study, we investigate the answers to these questions through the lens of shared reality theory and tend-and-befriend theory. Ultimately, we argue that shared reality reduces stressor reactivity during co-experienced stressors, and that this effect is moderated by sex.

Shared reality is the subjective experience of sharing an inner state—a thought, feeling, or concern—with another person (Echterhoff et al., 2009; Higgins, 2019). It reflects people’s tendency to turn to others to understand what is real, especially about things in the external world. According to Echterhoff et al. (2009), shared reality occurs when all four of its conditions are met:

The first condition is that a person must experience a subjective sense that their inner state aligns with someone else’s. Applied to stressful situations, two people would share reality if they spontaneously appraise a co-experienced stressor in the same way (Lazarus, 1993). Shared reality would not occur when two people only correspond in their outer states, such as when they mimic each other’s facial expressions during stressful events (Prochazkova and Kret, 2017) but do not actually align in their feelings about it.

Those shared inner states must refer to a specific target referent; the second condition (aka the “aboutness principle”) must be met (Echterhoff et al., 2009). Shared reality would not occur if two people simply align in their physiological stress (Timmons et al., 2015) or anxious moods (Berg et al., 2011; Ruffman et al., 2017; Frenzel et al., 2018) in the absence of an external stimulus. Instead, shared reality can only occur if people align in their interpretation of the same situation. Existing stress scholarship emphasizes the difference between stressors—concrete events in people’s environments—and stressor reactivity—people’s reactions to those events (Epel et al., 2018). Therefore, the stressors themselves are situations about which people could create shared realities.

The question then becomes whether people *want* to create shared realities in stressful circumstances. Is the third condition of proper motivation met? Research dating back to the beginning of social psychology suggests that it is. Inspired by Festinger’s social comparison theory (1957), Schacter ran a series of studies investigating whether and why females awaiting a shock experiment prefer to wait with other females awaiting the same fate (Schachter, 1959). Schacter consistently found that females prefer to wait together—what he termed the affiliation tendency—and also investigated the motives that underlie that preference. In his view, the affiliation tendency could be explained by a need to understand the situation (a drive for cognitive clarity), a need to understand one’s own emotions (a drive for self-evaluation), or a need to feel better (a drive for anxiety reduction). While Schacter found consistent evidence that females prefer to wait together rather than alone, the motives underlying the affiliation tendency have since failed to replicate (Cottrell and Epley, 1977; Kulik and Mahler, 2000).

In our view, Schacter and others’ inconsistent findings can be explained by two motivation tenets of shared reality theory: (1) that multiple motives can simultaneously underlie human behavior and (2) that social motives matter. To the first point, Schacter and his successors’ work implicitly assumed that only one motive could drive the affiliation tendency. They used experimental design to isolate each motive, with one condition fulfilling the drive for cognitive clarity, another condition fulfilling the drive for self-evaluation, and another condition fulfilling the drive for anxiety reduction. But according to shared reality theory, social interactions are driven by many motives that are not mutually exclusive nor fully understood by the existing scientific literature (Echterhoff et al., 2009). Instead, multiple cognitive motives fall under the broad umbrella of epistemic motives, which are the class of drives that lead people to make meaning of situations and establish valid understandings of them (Higgins, 2019). The drive for cognitive clarity, self-evaluation, and other cognitive processes can therefore be thought of as the constellation of motives that lead people to know the “truth” about a target as well as “what is valid” about their interpretations of it. In the context of co-experienced stressors, shared reality theory implies that multiple cognitive motives could simultaneously motivate a person to affiliate during co-experienced stressors.

Moreover, shared reality theory emphasizes that people are inherently social beings who are driven by a desire to be with and connect with other people. This drive is present in adjacent literatures on social support, empathy, and social sharing. For example, Rimé (2009) assert that people’s desire to talk about their emotions after an emotion-eliciting event is driven not only by a need for validation and legitimization (similar to epistemic motives), but also by a need for bonding and social sharing (similar to relational motives). Relational motives have also been shown to motivate the need to self-regulate in the aftermath of stressors (Zee et al., 2020) as well as during them (Ochsner, 2019; Zaki, 2020). However, this prior scholarship has only examined situations where one person is implicated in the stressor. As we will discover, social motives also underlie the desire to affiliate during co-experienced stressors. Taken together, the third condition of shared reality can be met during co-experienced stressors.

Having established that co-experienced stressors constitute opportunities for shared inner states (condition 1), that they are concrete events that could trigger a shared reality experience (condition 2), and that people can be properly motivated to create shared realities during them (condition 3), we turn to the fourth and final condition of shared reality theory: that the need for a shared reality is actually fulfilled. Such fulfillment could have important consequences for stressor reactivity during co-experienced stressors. As we discussed before, shared reality fulfills epistemic and relational motives. Because of this, shared reality could make people less reactive to co-experienced stressors.

This hypothesis is consistent with evidence suggesting that fulfilling epistemic and relational motives via shared reality brings positive outcomes. Cognitively, creating a shared reality with another person or group of people enhances memory (Echterhoff et al., 2005) and reduces cognitive dissonance (Rossignac-Milon and Higgins, 2018a). From a relational perspective, constructing a shared reality improves relationship maintenance between two people (Rossignac-Milon and Higgins, 2018b) and plays a larger role in explaining relationship outcomes compared to other important constructs such as commitment, intimacy, trust, and perceived similarity (Rossignac-Milon et al., 2021). Given the positive epistemic and relational outcomes associated with shared reality in other contexts, and given the motivational need to gain truth and bond with others during stressors (see above), it seems likely that successfully creating a shared reality with another person could reduce people’s reactivity to a stressor, relative to when two people fail to create a shared reality while jointly facing the same stressful situation. Moreover, the extensive literature on social support, interpersonal emotion regulation, empathy, shared emotional experiences, and mere presence implies beneficial effects of another person in the stress process. Taken together, shared reality during co-experienced stressors might reduce stressor reactivity.

However, a newer body of work suggests otherwise. According to a burgeoning literature by Boothby et al. (2014, 2016), shared experiences are amplified. The theory states that co-attending to the same stimulus as another person increases the prominence of that stimulus, which generates larger psychological and experiential responses regardless of the valence of the stimulus. Therefore, when a person eats sweet chocolate in the same room as another person, the chocolate states sweeter relative to when the person eats it alone in a room. The same phenomenon occurs for the bitterness of bitter chocolate. The corollary is that co-attending to the same stressful situation could inflate the prominence of the stressor, which could amplify stress reactivity. This hypothesis is in stark contrast to the one posed by shared reality that we outlined above. In our studies, we test between these competing hypotheses by running two-tailed statistical tests for the effect of shared reality on stressor reactivity. However, conceptually, we find the amplification process unlikely, especially in light of hypotheses generated by tend-and-befriend theory.

According to tend-and-befriend theory (Taylor et al., 2000), stressor reactivity is shaped by biological sex.^1^ Specifically, males exhibit ‘fight or flight’ during threat. During fight or flight, sympathetic arousal is accompanied by a series of hormonal reactions that drive males to use strength to save themselves. This makes sense from an evolutionary perspective, as warding off or effectively escaping from a threat increases the probability of survival. In contrast, fighting or fleeing would not be adaptive for females, who are instead responsible for both themselves and their offspring. Rather than fight a threat who could be stronger, or flee and leave their offspring behind, females ‘tend’ to offspring and ‘befriend’ other females to survive threats. Interestingly, a different biological mechanism underlies this tendency; females exhibit higher levels of oxytocin during stressful encounters. Because oxytocin enhances relaxation, reduces fearfulness, and decreases sympathetic activity, it enables mammals to return to homeostasis in the aftermath of stressors (Costa-e-Sousa et al., 2005). The fact that females exhibit higher oxytocin levels during stressors (Jezova et al., 1996) implies that females have a counter-regulatory system that helps them tend to offspring and befriend other females. Most importantly, oxytocin is associated with greater levels of social bonding (Uvnäs-Moberg, 1998; Feldman, 2012), which is why oxytocin is considered the biobehavioral mechanism underlying the tend-and-befriend response in females.

With this in mind, females are more likely to create and benefit from shared reality during co-experienced stressors. Consider that coordinated action during a stressful situation depends on females seeing the situation in the same way. If one female viewed a situation as highly stressful and the other viewed it as less so, there would be no way for them to deal with the stressor effectively. Or if one female appraised a situation as stressful but the other provided no indication that they felt the same way, the initial female would have less confidence, as the bond between them would be uncertain. But if both females agreed about the stressfulness of the situation—if they created a shared reality about the stressor—then they could easily jump into action. This would be driven by the fulfillment of epistemic and relational motives, enabling them to gain cognitive clarity about the meaning of the stressor, evaluate their own feelings about it as valid, as well as confirm their relational bond. Therefore, the benefits of affiliating while stressed could emerge for females who successfully create shared realities during co-experienced stressors.

Outside of evolutionary and biological explanations, which are hard to test empirically, there are developmental and role differences that could drive different effects of shared reality on stress reactivity for males and females. Throughout development, males are often taught to suppress their emotions while females learn to communicate and express how they feel (Brody, 1985). These developmental differences partially explain why females are more emotionally sensitive in stressful contexts; females are more susceptible to stress-related emotion contagion than males (Wild et al., 2001), more frequently provide emotional support after stressors (Joo et al., 2020), and behave more cooperatively and pro-socially than males once a stressor is over (Nickels et al., 2017). It follows logically that females would be more likely to create shared realities during co-experienced stressors than males and that they might also benefit from them.

Nonetheless, sex—explained evolutionarily, biologically, and socially—likely does not fully differentiate the effect of shared reality on stressor reactivity. To be clear, Taylor et al. (2000) do not view tend-and-befriend as prescriptive nor deterministic. Tending and befriending is instead considered a central tendency for females, and culture and gender roles may interact with those biological tendencies. Moreover, theories of socialization and gender do not perfectly predict individual differences on any outcome, including stressor reactivity. Nonetheless, no research to date has taken these claims, and instead has either ignored tending and befriending among males or has looked only for average sex differences in the tendency to affiliate under stress or not. This is likely because common methodological tools in the biological and social sciences reflect central tendencies only; the propensity for the average female to respond in one way and the average male to respond in a different way. We are the first to investigate Taylor et al.’s (2000) hunch; we leverage dyadic daily diary data to determine the percent of females and the percent of males who show stress reduction when experiencing shared reality during co-experienced stressors. This approach can paint a fuller picture of the importance of biological sex in shaping the effects of shared reality on stressor reactivity. We will be able to empirically acknowledge that biological mechanisms are not deterministic, but instead interact with individual differences regardless of sex assigned at birth. From there, we can theorize about the reasons why some females may not benefit from shared reality during a stressor and why some males may show prototypical female responses.

## Transparency and Openness

For both studies, we specify how we determined our sample size, manipulations, measures, and other standards set forth in the Open Science movement (see Appelbaum et al., 2018). All data, data environment information, code, and research materials are available at our OSF repository: https://osf.io/t7gdf/. Our studies were not pre-registered because this was preliminary, exploratory research. We discuss the implications of non-pre-registration in the discussion section.

## Study 1

Our first study contains only females; undergraduates who are asked to give a speech in front of a panel of evaluators alongside a confederate. Shared reality is manipulated through the presence or absence of social validation (see Hardin and Higgins, 1996). The confederate either (a) views the speech in the same way as the participant (shared reality confirmed), (b) views the speech in the opposite way (shared reality disconfirmed), or (c) does not provide an appraisal of the stressor (ambiguous). All confederates were females in order to increase the likelihood of success of our manipulation; shared reality is theorized to be strongest among ingroup members (Echterhoff et al., 2013).

We measure participants’ psychological stress immediately before the speech via self-report and physiological stress throughout the speech via an electrocardiogram and impedance cardiography, which together provide indices of self-reported stress, autonomic arousal, sympathetic arousal, and parasympathetic arousal. This design enables us to test whether shared reality reduces stressor reactivity compared to situations where the opportunity for a shared reality is denied.

### Participants

We recruited one hundred and eleven students through the Columbia University subject pool to complete the study for course credit (three of six credits) between March and June 2019. In the Columbia psychology subject pool, students choose a study from a list of anonymized options, and researchers are required to run all participants who have signed up for their study. Thus, we randomly sampled from the population of Columbia undergraduate students enrolled in an introductory psychology course. Through this method we collected data from both male and female participants but were only interested in the effects for female students due to our hypothesis that shared reality will reduce stressor reactivity for females. Therefore, we only consider data for the 79 female students who completed the study, which translates to 77% of the available sample. An additional six of those females were excluded from analyses due to meeting other exclusion criteria, including explicitly mentioning suspicion of the confederate (i.e., “the other participant was planted”) and/or being overtly suspicious of the stressful speech task (i.e., “the judges were not actually judging me”). Finally, three participants either did not complete relevant psychological questionnaires due to administrative errors or did not have usable physiology data. Therefore, the final sample size for the psychological and physiological analyses presented below is either 73 or 70, depending on which data was missing for the participant.

Of those included in the analyses, 47% identified as White, 29% identified as Asian or mixed Asian, 18% identified as Black/African American or mixed Black, and the remainder identified as other, other mixed-race, or American Indian/Alaska Native. Eighty one percent of the participants were psychology majors and the mean age was 20 years (*SD* = 2 years).

### Procedure

**FIGURE 1:**
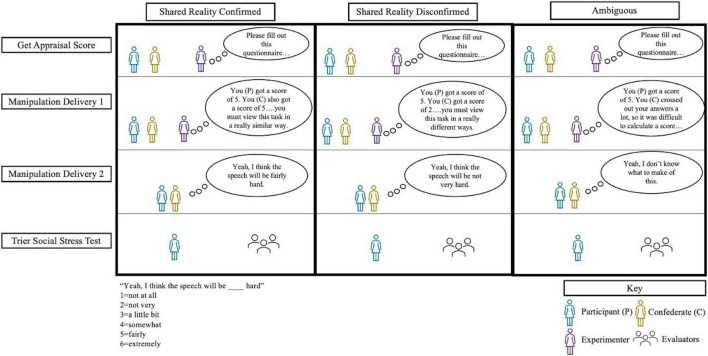
Schematic of study procedures.

A schematic of the manipulation can be found in Figure 1. The confederates and experimenters were all volunteer research assistants and were matched to participants according to gender (since only data from female participants is used here, all confederates were female). Scripts containing the exact training and study procedures can be found on our OSF repository^2^ while a summary can be found here.

Upon arrival, participants met a confederate in the lobby and were greeted by an experimenter. The confederate and the participant were brought to the same room, where they were apprised of study procedures. Participants consented to be in the study about (1) “physiological activity during rest vs. during more active tasks” and (2) “to examine the relationship between physiological responses and individual differences based on questionnaires.” During debriefing, participants were told full information about the purposes of the study. Participants were never explicitly told about the experimental condition they were in.

The confederate was then “randomly” selected to go to a separate room so that both could be fitted with physiological sensors for electrocardiography and impedance cardiography. Participants’ baseline physiology was measured for 5 min as they relaxed in a chair by themselves. Following baseline and an unrelated physiology task, the confederate was brought back into the participant’s room and seated in an armchair next to the participant. Both the participant and the confederate were then introduced to the Trier Social Stress Task (TSST; Kirschbaum et al., 1993). The TSST is the ‘gold standard’ stress induction paradigm due to its successful elicitation of heightened cortisol levels, heart rate, and skin conductance (Goodman et al., 2017). It involves giving an impromptu speech to a panel of evaluators who are ostensibly trained in judging non-verbal behavior. After being introduced to the stress task, participants completed a validated appraisal questionnaire indicating how demanding they viewed the speech task, with an average of 1 indicating ‘not at all demanding’ and an average of 6 indicating ‘extremely demanding’ (Mendes et al., 2001). The experimenter left the room for 1 min to compute the participant’s appraisal score. Participants were then randomized into one of three conditions.

In all conditions, the experimenter read out loud the participant’s true appraisal score from 1 to 6. In the shared reality confirmed (SRC) condition, the confederate “got the same score” as the participant, i.e., if the participant viewed the speech as a 5 the confederate viewed the speech as a 5. In the shared reality disconfirmed (SRD) condition, the confederate “got the opposite” score as the participant, i.e., if the participant viewed the speech as a 5 the confederate viewed the speech as a 2. The opposite score was calculated as 7 minus the participant’s score. This means that if the participant viewed the speech below the mid-point of the scale, i.e., a 2, then the confederate had a score that was as extreme but on the opposite end of the scale, i.e., a 5. However, if the participant viewed the speech above the mid-point of the scale, i.e., a 5, then the confederate had an equally extreme but lower score, i.e., a 2. Conceptually, we view both of these forms of the manipulation to be explicit rejections of shared reality, in which the confederate got a score as extreme but on the other end of the appraisal questionnaire as the participant. Interested readers should refer to a secondary analysis we conducted in the Supplementary Materials that confirms that the results hold regardless of whether the confederate was more or less stressed than the participant. In the ambiguous (AMB) condition, the confederate crossed out their answers so much that they ‘‘did not provide a use-able’’ appraisal score. There were 26, 23, and 24 participants in the SRC, SRD, and AMB conditions, respectively.^3^ To further deliver the manipulation, the experimenter made an “ad-libbed” comment that appeared to deviate from the script where they either noted the similarity (SRC) or difference (SRD) in scores between the confederate and the participant. In the AMB condition, the experimenter did not make an additional ad-libbed comment. The experimenter then left the room to check on the physiology signals. During the few moments that the experimenter was out of the room, the confederate delivered a line that re-iterated to the participant that they either felt the same, felt differently, or just didn’t know how to feel.

Immediately after the manipulation (but before the speech), the confederate was brought to a separate room, ostensibly to perform their own speech. This meant that the participant was not with the confederate when giving the speech to the panel of evaluators. During this intermediary time, the participant filled out a brief questionnaire with a manipulation check, a measure of cognitive clarity, and a measure of self-reported psychological stress. After this short questionnaire, the participant gave the speech and physiological stressor reactivity was continuously measured through electrocardiography and impedance cardiography to provide an unobtrusive, non-conscious, and continuous measure of stress during the speech. Following the speech, the participants completed another questionnaire including items about relational closeness.

### Measures

In our view, it is worthwhile to test our research question in terms of both psychological and physiological stress, especially given the biological underpinnings of tend-and-befriend theory. Emerging consensus in the stress field emphasizes the importance of multimethod approaches to stress research (Epel et al., 2018). This is because different measures each have their strengths and weaknesses, as well as the fact that they map onto separate components of the stress response (Mauss and Robinson, 2009). Self-report measures of psychological stress are valid insofar as they reflect people’s conscious, subjective, and experiential responses to a situation. Self-reported stress before and after a stressor predicts critically important psychological outcomes, such as well-being (Almeida, 2005), relationship satisfaction (Randall and Bodenmann, 2017), and subjective age (Kotter-Grühn et al., 2015; Bellingtier et al., 2017). We therefore measure psychological stress via self-report immediately after the shared reality manipulation but before the stressor. Nonetheless, self-reported stress is limited in that it contains measurement error due to people being unaware and/or incapable of reporting on their own stress levels, as well as the fact that self-reported measures are discrete in time.

Physiological stress measurement therefore provides a useful complement (Blascovich et al., 2011). Physiological stress reactivity enables the continuous measurement of stress before, during, and after a stressor. It is unbiased by a person’s level of emotional awareness or willingness to communicate with a researcher about how they feel. And perhaps most importantly for this study, measuring physiological reactivity enables us to break down stress into autonomic, sympathetic, and parasympathetic arousal.

Specifically, arousal in response to a stressful situation can be measured by heart rate reactivity, which represents the autonomic nervous system. The autonomic nervous system is composed of both the sympathetic and parasympathetic sub-systems. The sympathetic system is thought of as the ‘fight or flight’ system while the parasympathetic system is thought of as the ‘rest and digest’ system. Therefore, we can investigate whether shared reality reduces overall stressor reactivity and whether any differences are driven by physiological differences in ‘fight or flight’ versus ‘rest and digest.’ This will be especially interesting in light of tend-and-befriend theory, which concerns hormonal differences between fight or flight (limited oxytocin) and tend and befriend (high oxytocin) responses. Those hormonal differences in stress reactions map on to physiological differences insofar as oxytocin is linked to parasympathetic activity. As such, we expect that shared reality will increase parasympathetic arousal due to the tend-and-befriend hypothesis that oxytocin calms females down during stressors, and physiological states of calm correspond to parasympathetic activity.

### Self-Report Measures

A disattenuated correlation matrix for all psychological variables can be found in our Supplementary Materials.

#### Manipulation Checks

Following the shared reality manipulation but before the speech, participants completed an in-house measure of target shared reality. Items from this scale were: (1) *I think that the other participant and I are on the same wavelength with regard to the speech*, (2*) I feel the same way about the speech as the other participant*, (3) *I agree with the other participant’s point of view of the speech*, (4) *The other participant and I see the speech in the same way*, and (5) *I agree with the other participant’s perception of the speech.* Cronbach’s alpha was 0.94. Scale items ranged from 1 = Strongly disagree to 7 = Strongly agree and an average was computed for each participant. This scale has been used in prior research (see Rossignac-Milon et al., 2021). Because this scale measured perceived shared reality, participants in the SRC condition should have higher scores than those in the SRD condition. Since participants in the AMB condition indicate that they are unsure how to feel, there should be lower levels of shared reality in this condition relative to SRC. *A priori* we do not know whether participants in the AMB condition will have higher shared reality than those in the SRD condition. To our knowledge, no study has tested people’s assumptions about shared reality with another person when the other person expresses uncertainty. We assume the average shared reality in the AMB condition could be lower than the average in the SRC condition, but maybe not significantly so. Moreover, average shared reality in the AMB condition could be higher or perhaps even lower than the SRD condition, depending on whether participants interpret uncertainty as someone being the same or opposite from them or somewhere in between.

To confirm that higher shared reality fulfilled epistemic motives, we asked participants how strongly they disagree or agree with the following items on a 1–7 scale: (1) *I am uncertain about my perception of how demanding the speech is* (reversed) and (2) *I am sure that my impression of the speech is valid*.^4^ These items were generated in-house, with a Cronbach’s alpha of 0.76. Averages were computed for these three items and participants in the SRC condition should have higher scores than those in the AMB and SRD conditions. To confirm that higher shared reality fulfilled relational motives, we asked participants how much they strongly disagree or agree with the following items from Ryan’s (1982) relatedness scale, from −7 scale: (1) *I feel really distant from the other participant* (reversed), (2) *I really doubt that the other participant and I would be friends* (reversed), (3) *I feel like I could really trust the other participant*, (4) *I’d like a chance to interact with the other participant*, (5) *I’d really prefer not to interact with the other participant in the future* (reversed), (6) *I don’t feel like I could really trust the other participant* (reversed), (7) *It is likely that the other participant and I could become friends if we interacted a lot*, (8) *I feel close to the other participant*. Cronbach’s alpha was 0.80. Averages were computed for these items and participants in the SRC condition should have higher scores than those in the AMB and SRD conditions.

#### Pre-speech Self-Reported Psychological Stress

Following the manipulation and before giving the speech, participants reported on their subjective stress via the anxiety subscale of the Profile of mood states (McNair et al., 1981). This is a common operationalization of psychological stress. Items include *nervous*, *anxious*, *panicky*, and *worried*. Participants rated each item on a scale from 0 = Not at all to 4 = Extremely and averages were computed for each participant, and Cronbach’s alpha was 0.83.

### Physiological Measures

Cardiac physiology was recorded non-invasively following established guidelines from the Society for Psychophysiological Research (e.g., Sherwood et al., 1990). We measured electrocardiography (ECG) and impedance cardiography (IMP) using Biopac’s ECG and NICO modules, respectively, which were integrated via the MP150 system. ECG was sampled at 1000 Hz with a modified lead II configuration, such that sensors were placed below the sternum and the left side of the torso below the ribcage. IMP was also sampled at 1000 Hz with band electrodes that that completely encircled the participant’s neck and torso. These collection methods follow Blascovich et al.’s (2011) guidelines.

The physiological signals were scored in 1-min ensemble windows with default algorithms in the Mindware software. Scoring of physiology signals relies partly on research assistants’ subjective decisions while manually checking and altering (if necessary) the Q, P, R, S, and T inflection points on the cardiac waveform. This is because the Biopac and Mindware software are imperfect in terms of detecting these inflection points. Of course, manual changes introduce potential biases in scoring, which could influence subsequent results. To gain confidence that little to no biases emerged from scoring, we ran reliability analyses using the full dataset with complete and usable physiology signals (102 participants). We used all 20 min of physiological data throughout the study, including 5 min of baseline, 3 min of preparation for the stress task, 2 min of the manipulation, 5 min of the TSST and 5 min of recovery. Two research assistants independently identified inflection points on all ECG and IMP signals and we ran ICC analyses to compute reliability. Results suggest that heart rate (ICC = 0.89, CI = 0.88–0.90), pre-ejection period (ICC = 0.87, CI = 0.85–0.88), and root mean square of successive differences (ICC = 0.84, CI = 0.83–0.85) were reliably scored.

#### Baseline

Once the experimenter applied all sensors to the participant, the participant was seated in an armchair and asked to relax for 5 min. The experimenter left the room and monitored the participant and physiology signals during this 5-min period.

#### Heart Rate Reactivity

Heart rate reactivity is an unobtrusive indicator of autonomic nervous system activation, which reflects stressor reactivity (Blascovich et al., 2011). Heart rate reactivity was computed as the difference between each participant’s average heart rate in the first minute of the speech, while reactivity is likely highest, and the final minute of baseline, when restfulness is likely highest. These difference scores were 95% winsorized, which is common in physiological data analysis due to the fact that extreme values likely result from noise in the data collection process.

#### Pre-ejection Period Reactivity

The autonomic nervous system consists of the sympathetic and parasympathetic nervous system. The sympathetic nervous system reflects the ‘fight or flight’ response and can be measured via pre-ejection period (PEP), which reflects cardiac contractility or contraction of the heart muscle (Blascovich et al., 2011). PEP is calculated by summing electromechanical delay (which comes from the Q wave on the electrocardiogram) to the onset of pressure rise in the left ventricle. For this index, more contraction results in lower values of PEP, meaning that greater levels of ‘fight or flight’ correspond to greater reductions in PEP.

To compute reactivity, difference scores were computed by subtracting the participant’s average PEP in the final minute of baseline from the average PEP in the first minute of the speech. These difference scores were 95% winsorized, which is common in physiological data analysis due to the fact that extreme values likely result from noise in the data collection process.

#### Root-Mean-Square of Successive Differences Reactivity

The other branch of the autonomic nervous system, the parasympathetic nervous system, reflects the ‘rest and digest’ response and can be measured via root-mean-square of successive RR differences (RMSSD) collected via impedance cardiography (Blascovich et al., 2011). RMSSD is calculated by measuring each successive time difference between heartbeats. Then, each successive time difference squared, an average of these squared differences is obtained, and the square root is taken. Since this measure reflects activation of the rest and digest system, we consider differences in the *reduction* of RMSSD across conditions.

To compute reactivity, difference scores were computed by subtracting the participant’s average RMSSD in the final minute of baseline from the average RMSSD in the first minute of the speech. These difference scores were 95% winsorized.

### Statistical Approach

We implement several ANOVA/regression models with Bayesian estimation, in which condition is the independent variable and various psychological and physiological outcomes are dependent variables. Because we recognize that the traditional approach to modeling this type of data would be to use frequentist estimation in one-way ANOVAs, we would like to point out that the parameter estimates are the same in both cases because we use non-informative priors in our Bayesian estimations.

We use Bayesian estimation because it enables us to make direct probability statements about hypothesized effects in our models (Van De Schoot et al., 2017). This is because Bayesian models give rise to posterior distributions, which represent the distribution of possible values for model parameters. In our models below, the posteriors reveal the distribution of potential values for the mean difference between groups. Because the mean difference itself has a distribution, the posterior, we can look at its mean, median, or mode to see the most likely value of the difference while also visualizing the spread around those values of central tendency. We can also compare the mean difference to a conceptually meaningful number such as 0 and compute the percent of the distribution that falls above or below that point (i.e., no difference). In simple terms, we can determine the probability that the mean is above or below zero.

By contrast, in frequentist statistics, none of these probabilities can be calculated. Instead, a frequentist approach typically evaluates how extreme (i.e., how far from zero) the observed study’s parameter estimates are relative to what might be expected were the study to be carried out an infinite number of times. If the observed estimates are improbable (i.e., *p* < 0.05) under the assumption of zero effect, one usually declares the results to be statistically significant. Thus, frequentist probability statements are about how unusual the observed data are compared to other possible datasets that could have been observed. There is nothing probabilistic about the parameters themselves.

Most detrimentally, in the case of frequentist confidence intervals, one cannot be confident in the intervals observed in a given study; one can only be confident in the long-run behavior of the procedure of calculating confidence intervals. Concretely, one cannot have 95% confidence that a particular interval will include the true parameter value; rather, one can be confident that 95% of the intervals obtained from identical studies—were such studies to be conducted–would include the true value. Our use of Bayesian estimation instead allows us to think probabilistically, which aligns with rising concerns about binary significance testing because it encourages us to think distributionally rather than in binary terms (Wagenmakers, 2007; Dienes, 2011).

Nonetheless, cutoff values can decrease cognitive load as readers go through the results. If there is an 85% probability that the difference is above zero, do we conclude that a difference has emerged? What about 80%? We choose the value of 90% probability that the mean is above (or below) zero to make statements about differences in a binary sense. We chose this value because it is the point at which a visible amount of the distribution can be seen in graphic representations of the posterior distributions. In terms of Bayes Factors (BF), this translates into a BF of 9. In our view, the visual representations are superior to BFs because BFs can also encourage binary thinking. Thus, while we set a cutoff rule of at a BF of 9, we encourage readers to interpret our results visually and distributionally rather than in binary terms.

## Results

Visualizations of the raw data for all dependent variables can be found in the Supplementary Materials.

### Manipulation Checks

**FIGURE 2:**
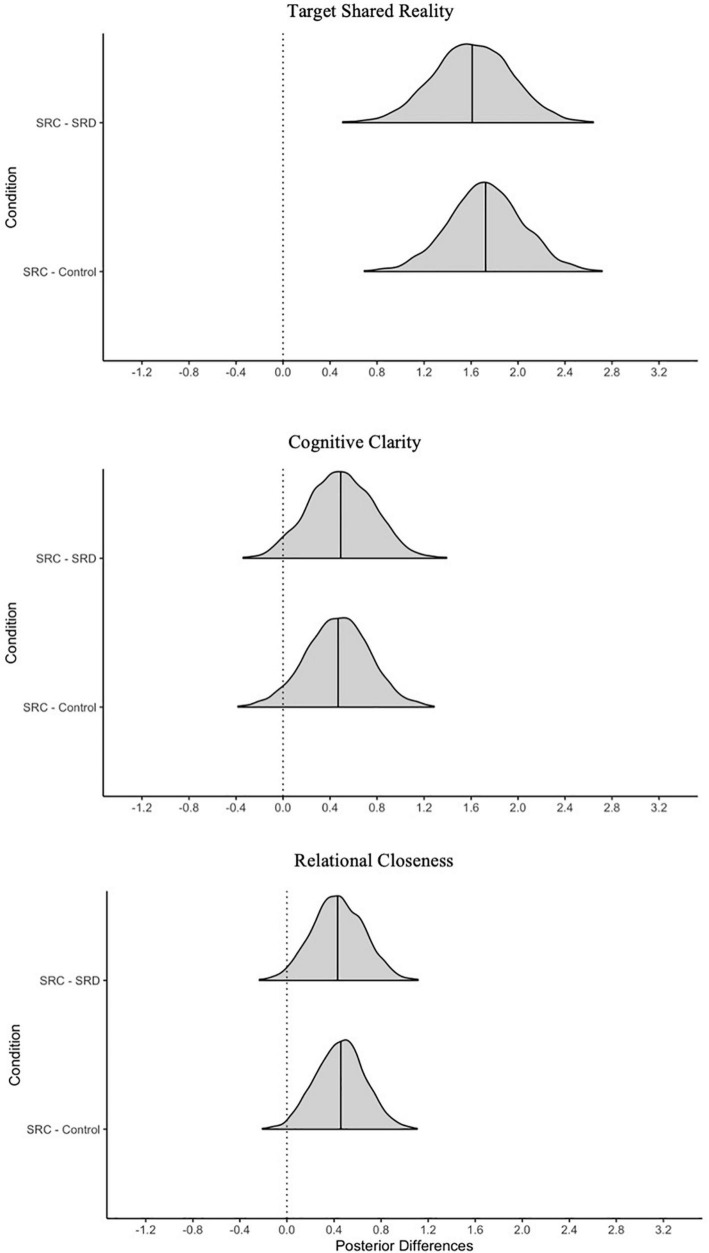
Posterior differences in manipulation checks by condition. SRC, shared reality confirmed; SRD, shared reality disconfirmed; AMB, ambiguous. Solid line represents the 50th percentile and the dotted line on 0 = no difference.

Visualizations of all manipulation checks can be found in Figure 2.

#### Shared Reality

The first and most crucial manipulation check concerns the shared reality measure. Did participants in the SRC condition feel a greater sense of shared reality with the confederate than those in the disconfirmed condition? And how did shared reality differ between the SRC and AMB conditions? As hypothesized, participants in the SRC condition (*M* = 5.7, *SD* = 1.5) had higher shared reality with the confederate than those in the SRD condition (*M* = 3.8, *SD* = 1.4). As Figure 2 shows, essentially all posterior values of the difference between these means exceed zero, leading us to be very confident that the SRC condition elicited more shared reality than the SRD condition. Shared reality was also higher in the SRC relative to AMB condition: The difference between the SRC and AMB condition (*M* = 3.8, *SD* = 1.1) was positive and of a similar magnitude.

#### Epistemic Motives Fulfilled

We next consider differences in the extent to which epistemic motives were fulfilled between participants in each condition. As expected, participants in the SRC condition had greater levels of epistemic fulfillment about the stressor (*M* = 5.0, *SD* = 1.2) than those in the SRD condition (*M* = 4.0, *SD* = 1.5). The posterior probability of a positive difference was greater than 99%. Epistemic motives were also more fulfilled in the SRC relative to AMB condition (*M* = 4.0, *SD* = 1.0) with more than 99% of the posterior differences being greater in the SRC condition. We again conclude that epistemic motives were more fulfilled in the SRC relative to AMB condition.

#### Relational Motives Fulfilled

Our final manipulation check concerns relational closeness or the fulfillment of needs for relational bonding during the stressor. Results suggest that participants in the SRC condition (*M* = 4.7, *SD* = 0.9) felt greater relational closeness with the confederate than those in the SRD condition (*M* = 3.9, *SD* = 0.9). There was more than a 99% chance that relational closeness was higher in the SRC condition compared to the AMB condition. Similarly, relational closeness was reported to be greater in the SRC relative to AMB (*M* = 3.9, *SD* = 0.9) conditions with more than a 99% of the mean differences in this direction.

### Self-Reported Psychological Stress

**FIGURE 3:**
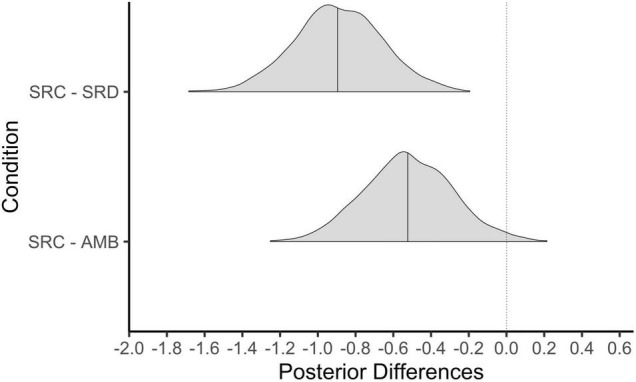
Posterior differences in psychological stress by condition. SRC, shared reality confirmed; SRD, shared reality disconfirmed; AMB, ambiguous. Solid line represents the 50th percentile and the dotted line on 0 = no difference.

Now that we have established that shared reality and both types of motives were fulfilled in the SRC condition relative to the SRD and AMB conditions, we investigate whether those differences in shared reality and its concomitant motives correspond to differences in self-reported anxiety. Results show that participants in the SRC condition (*M* = 2.2, *SD* = 0.7) were less stressed than those in the SRD condition (*M* = 3.1, *SD* = 0.9). As Figure 3 shows, essentially all posterior values of the difference between these means were less than zero, leading us to be very confident that the SRC condition elicited less psychological distress than the SRD condition. Participants also reported being less stressed in the SRC relative to the AMB condition (*M* = 2.7, *SD* = 0.8). Here, the posterior probability of a negative difference was 98%.

### Physiological Stress

Having established that shared reality is greatest in the SRC condition, and that self-reported psychological stress in the moment before the speech is also lowest in that condition relative to both the SRD and AMB conditions, we investigate whether the same is true for physiological stress during the speech, which is an unconscious and unbiased measure of stress reactivity.

#### Autonomic Nervous System (Overall Physiological Stress): Heart Rate Reactivity

**FIGURE 4:**
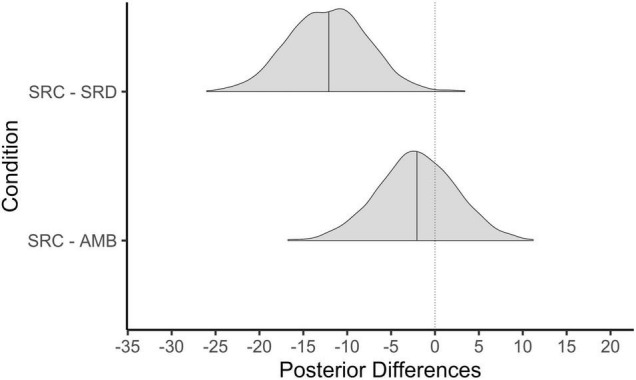
Posterior differences in heart rate by condition. Solid line represents the 50th percentile and the dotted line is 0 = no difference.

As with psychological stress, results suggest that participants in the SRC condition were less stressed during the speech (*M* = 23.7, *SD* = 13.0) than those in the SRD (*M* = 35.9, *SD* = 15.7) condition. The probability of this was more than 99%. Diverging from the self-reported results for psychological stress before the speech, we were less sure of the difference between participants in the SRC and the AMB (*M* = 25.8, *SD* = 16.6) conditions. Here, there was a 67% probability that participants in the SRC condition were less stressed than those in the AMB condition (see Figure 4).

#### Sympathetic Nervous System (‘Fight or Flight’): Pre-ejection Period Reactivity

**FIGURE 5:**
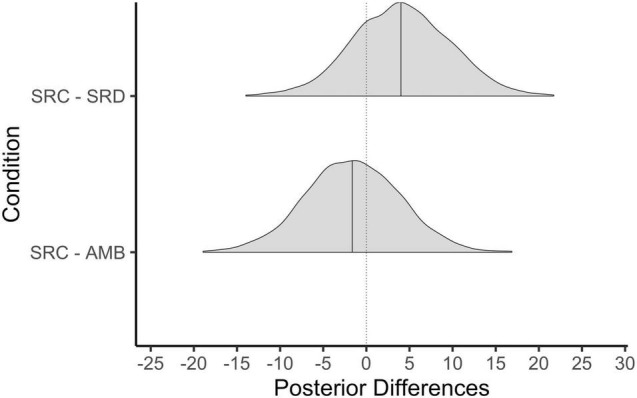
Posterior differences in PEP (‘Fight or Flight’) by condition. Solid line represents the 50th percentile and the dotted line is 0 = no difference.

Results for the fight or flight system are uncertain and, overall, do not allow us to conclude that differences in ‘fight or flight’ activity emerged between conditions. When interpreting the means remember that lower PEP corresponds to greater activation of the ‘fight or flight’ system. Results suggest that participants in the SRC condition (*M* = −30.8, *SD* = 20.2) had higher PEP than those in the SRD condition (*M* = −34.9, *SD* = 17.7). However, there was only a 76% chance of this. There was also a small indication that participants in the SRC condition had greater PEP than those in the AMB condition (*M* = −29.25, *SD* = 18.3), but there was only a 38% chance of this (see Figure 5).

#### Parasympathetic Nervous System (‘Rest and Digest’): Root-Mean-Square of Successive Differences Reactivity

**FIGURE 6:**
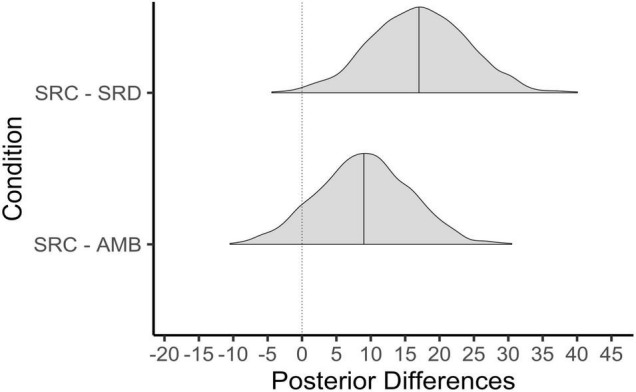
Posterior differences in ‘Rest and Digest’ by condition. Solid line represents the 50th percentile and the dotted line is 0 = no difference.

Results for the ‘rest and digest’ system are much more interpretable and together reveal how shared reality impacts stress, specifically its reduction. When interpreting these results remember that RMSSD is expected to be reduced in every condition, since every condition contains the stressful task. The comparison here is whether the shared reality condition elicits fewer reductions in RMSSD relative to the SRD and AMB conditions. Results show that participants in the SRC condition had fewer reductions in the ‘rest and digest’ system (*M* = −17.09, *SD* = 24.7) compared to those in the SRD condition (*M* = −34.1, *SD* = 22.2). The probability of this was 99%. Again, we were less sure of the difference between participants in the SRC and the AMB (*M* = −26.1, *SD* = 22.1) conditions. Here, there was a 90% probability that participants in the SRC condition were less stressed than those in the AMB condition. We cannot be fully confident (see Figure 6).

### Discussion

A summary of the results from Study 1 can be found in Table 1. These findings suggest that experiencing shared reality with another person about a stressor dampens psychological and physiological stress. Participants in the shared reality confirmed (SRC) condition reported being less anxious in the moments leading up to the stressor, relative to participants in the shared reality disconfirmed (SRD) and ambiguous (AMB) conditions. This is likely due to the combined power of greater epistemic certainty and relational closeness that participants felt in the SRC condition.

**TABLE 1:** Summary of results from Study 1.

	Model-predicted mean difference*	Finding	Probability
**Manipulation checks**			
Shared reality			
SRC > SRD	1.82	Yes	>99%
SRC > AMB	1.85	Yes	>99%
Epistemic motives fulfilled			
SRC > SRD	0.81	Yes	>99%
SRC > AMB	0.84	Yes	>99%
Relational motives fulfilled			
SRC > SRD	0.73	Yes	>99%
SRC > AMB	0.74	Yes	>99%
**Primary analyses**			
Psychological stress			
SRC < SRD	0.89	Yes	>99%
SRC < AMB	0.52	Yes	98%
Overall physiological stress			
SRC < SRD	12.17	Yes	>99%
SRC < AMB	2.05	Unsure	67%
Fight or flight			
SRC < SRD	4.07	Unsure	24%
SRC < AMB	1.60	Unsure	62%
Rest and digest			
SRC > SRD	17.00	Yes	99%
SRC > AMB	8.98	Yes	90%

*These are the means of the posterior distributions.

These psychological benefits of shared reality persisted as participants underwent the stressful task, as physiological stress was lowest in the SRC condition. Specifically, participants in the SRC condition exhibited lower levels of heart rate reactivity relative to those in the SRD condition. Our evidence comparing the SRC to AMB condition was less robust on this measure. When we investigated deeper, looking at sympathetic and parasympathetic arousal, we found that these physiological benefits of shared reality manifested as fewer reductions in the ‘rest and digest’ system. Participants in the SRC condition were better able to maintain parasympathetic arousal compared to participants in the SRD and AMB condition. We were surprised that no reliable results emerged in the sympathetic system, especially in light of prior findings that sympathetic arousal is lowest when oxytocin is released (Petersson et al., 1996; Camerino, 2009; Moberg et al., 2019). While we can conclude that shared reality buffers against the negative physiological effects of stress by increasing calmness via parasympathetic arousal, we are uncertain whether it also decreases fight- and flightiness. As a whole, Study 1 provides the first evidence to suggest that sharing reality about a stressor with another person is beneficial for psychological and physiological health, at least for females.

## Study 2

While Study 1 provided experimental evidence for the effects of manipulated shared reality on stressor reactivity for females, as well as the impact on psychological and physiological stressor reactivity, Study 2 generalizes those findings to non-university students navigating co-experienced stressors in daily life. Importantly, Study 2 not only investigates the effects of shared reality among females but also among their male romantic partners. Given that tend-and-befriend theory poses no hypotheses for the social psychology of stress for males, we consider our analysis of male-female interactions to be a sufficient first test for how males react to shared reality with other people during co-experienced stressors.

Study 2 is a 14-day dyadic daily diary study in which romantic couples living in New York City provide evening reports of stressors that they co-experienced with their partners, how much shared reality they felt during those experiences, and how stressed they were by the end of the day during the first year of the COVID-19 pandemic. Our stopping rule for data collection was reaching the pre-set limit of our research budget. That point also aligned nicely with lifted restrictions during the COVID-19 pandemic in March of 2021. We use a novel design in which a research team member meets with each couple prior to the diary period to provide detailed instructions. The research assistant asks couples to briefly co-identify a co-experienced stressor each evening before they fill out the diary survey, but to otherwise not discuss anything about the survey. After collecting the data, we found that participants typically agreed with their partner that the co-identified stressor was the ‘right’ choice (on a 1–5 scale, mean = 4.4, between-person *SD* = 0.6), and that dyad members generally identified the same event in a text response (99.3% of text entries matched between partners). Once couples identify the stressor, each member fills out the diaries separately. This methodology enables us to look at the effects of shared reality on self-reported psychological stress for each member of a dyad for a mutual co-experienced stressor.

Because of the intensive repeated measures nature of the data, we will be able to estimate not only the extent to which shared reality reduces stress reactivity for females and males on average but also how much variability there is in these slopes. A comprehensive conceptual description of this approach can be found in Goldring and Bolger (2021), so for now we briefly point out that random slopes in multilevel models give rise to an average slope between shared reality and stressor reactivity as well as a slope between shared reality and stressor reactivity for each person in the sample.

### Methods

#### Procedures

All study procedures were approved by the Institutional Review Board at Columbia University. Participants were recruited into a study “about current events influence stress and mental well-being [among]…romantic couples.” Thus, participants learned vague information about the study prior to participating and full information during debrief, where they were told that the purpose of the study “was to examine how daily stressors (both related and unrelated to COVID-19 and current social and political events) influence daily mood and well-being, as well as how romantic relationships are implicated in response to these stressful events.”

Data from this study comes from a larger project investigating the social and psychological effects of the COVID-19 pandemic among cohabitating romantic couples. Between August 2020 and March 2021, couples were recruited through online postings and crowdsourcing (e.g., Facebook, Craigslist, Twitter, Honeybee Hub, University Listserv), as well as flyers around New York City area and word of mouth. Every third Tuesday, a new cohort started; this ensured that all couples completed the diary from Tuesday to Tuesday (14 days apart) and enabled us to keep track of current events that might impact each cohort. Participants in the first 2 weeks received $20 compensation per participant; because this was low, the following cohort were incentivized with $30 per participant. The final two cohorts were incentivized with $40 per participant because recruitment had slowed down considerably by then. Eligibility criteria were: being above 21 years of age, in a cohabitating romantic relationship in which no other people live in the household, being in the New York City area for the duration of the study, and having consistent access to high quality internet. Eligibility was further contingent on both members of the couple completing a Zoom call and a baseline questionnaire, discussed next.

Three hundred forty-five couples were invited to jointly attend Zoom calls with a research assistant from the lab who explained study procedures, answered questions, and ensured that the members of the couple were in the same household and were not bogus responders. During the Zoom call, participants were given instructions pertinent to co-experienced stressors. They were asked to talk with one another briefly each night before completing the survey. In this brief meeting, they were told to identify a stressful event that they co-experienced that day. The instructions read:

“There is only one thing that we do ask you to discuss with your partner prior to completing the daily survey. Each day, you will be asked questions about one specific stressful event that you and your partner **both experienced.** For example, maybe something in your apartment broke, a mutual friend of yours was diagnosed with COVID, or a new policy came out that impacts both of you. What’s important is that the event is new and impacted both of you. **We ask that you briefly identify this event with your partner before filling out the survey each day.** If you did not get a chance to consult with your partner, we ask that the first person to complete the survey identify the new stressful event and tell their partner which event they chose.

We understand that on some days it may be difficult to identify a new stressful event that you both experienced. **That’s OK, just do your best to identify whatever was most stressful that you both experienced that day.**”

If participants did not co-experience a stressful event (i.e., they indicated ‘no’ to a stem question about whether or not they co-experienced a stressor), they answered the same set of questions about a different co-experienced event that they did not have to consult on, for example watching TV, cooking dinner, or anything else they wanted to report on.

One hundred sixty-five couples completed the Zoom call and were emailed baseline surveys measuring aspects of their relationships, personalities, and chronic stress environments. No baseline variables are included in the analyses presented here. At the individual level, three hundred nine people completed baseline, of which two hundred fifty-two were deemed eligible. Note that some people who were initially deemed eligible were still filtered out at this stage, either for indicating that they had children of an age that the child actually lived with them, not matching their participant on pertinent questions (for example, one member of the couple said they had been together for 8 years and the other said 2 years), or because their partner simply did not fill out the baseline survey. Once both partners completed the baseline questionnaires, they were deemed eligible for the diary portion of the study. There were one hundred twenty-six couples who entered the diary stage.

Each evening at around 7pm (range: 5pm–8pm), participants were sent an email with a link to the survey. The survey included a host of items, a subset of which are used in the analyses below (see Materials and Measures below). Most pertinently for the analyses below, participants were asked if they jointly experienced a stressful event that day. If they responded ‘yes,’ they were piped into a portion of the survey that asked relevant questions about the shared stressor. If they responded ‘no,’ participants were piped into a different portion of the survey where they were asked similar questions but about a co-experienced event that was not considered stressful. One hundred twenty-three couples filled out at least one diary entry. Following the 14-day diary period, participants received payment if they completed at least 11 diary entries. However, for the purposes of the analyses in this manuscript, all participants are included if they completed at least one diary entry. Following the diary period, participants were debriefed and thanked for their time.

Interested readers may refer to the Supplementary Materials so see a recruitment flowchart for this study.

#### Participants

A full flow cart of participants in the study can be found on our OSF repository (see text footnote 2). Because our analysis concerns biological sex, we subset the data to only heterosexual couples with one *cis*-male and one *cis*-female. The final sample size that met all of the above-mentioned criteria was 102 couples, 102 males and 102 females. Of those, 90 couples co-experienced at least one stressful event together. Participants were on average 28 years old (*SD* = 8), in their relationships for 4 years (*SD* = 3.9), and 33% were married. The racial/ethnic composition of the sample was 50% white, 26% Asian, 5% Black or African American, 8% mixed race, and 11% in an ‘other’ category; 17% identified as Hispanic/Latinx.

### Materials and Measures

#### Shared Stressors

Couples were asked (‘yes’ or ‘no’) whether they co-experienced a stressor. A ‘no’ response piped participants to a portion of the survey about a non-stressful co-experience, one that did not necessarily have to match between couple members. A ‘yes’ response piped participants to a portion of the survey containing a text box indicating what the shared stressor was. The definition of a shared stressor in the context of shared reality theory is that it must be an external event that the couples co-experienced together. Although participants spoke briefly with each other prior to completing the survey and determined if and what their shared stressor was, couples might have identified events that did not meet our criterion. Therefore, to confirm that the text responses were indeed external stressful events and that both members of the couple identified the same event, two coders blindly scored the text responses and resolved discrepancies by discussion. If both partners identified the same stressful event that was external to them and their relationships, the shared stressor variable was coded as a 1, and 0 otherwise. Prior to resolving discrepancies, the inter-rater reliability (computed as an intraclass correlation coefficient) on this measure was 0.82 with a 95% confidence interval of 0.80 to 0.83. On average, couples co-experienced three stressful events throughout the diary period (between-couple *SD* = 3, range = 0–12). Prior to analyses, this variable was disaggregated into between- and within-couple levels of analyses. Only the within-couple level is used here, as the within-couple level reveals stressor-by-stressor variation in the effect of shared reality on psychological stress (the between-couple would reveal whether couples who are higher in shared reality across stressors are also lower in psychological stress on average across all study days). Interested readers should refer to Bolger and Laurenceau (2013) for further details on within- versus between-units of analysis.

#### Subjective Shared Reality

A subset of the shared reality items that were used in Study 1 was used here. The three items were: As it was occurring, my partner and I seemed to… (1) *be on the same wavelength about [the stressor]*, (2) *feel the same way about [the stressor]*, (3) *have the same perception of [the stressor].* Participants responded on a 1–5 scale from 1 = *Strongly disagree* to 5 = *Strongly agree.* Here, we computed reliability of within-person change as a measure of reliability following guidelines by Bolger and Laurenceau (2013). We found that the scale had an R_*c*_ of 0.91. Averages of these three items were computed for each participant (*M* = 3.6, between-person *SD* = 1.0). Prior to analysis, these scores were separated into between- and within-person levels (see Bolger and Laurenceau, 2013). Only the within-person level of analysis is included in the analysis.

#### Psychological Stress

Just as we operationalized subjective stress as anxiety in Study 1, we use the anxiety subscale of the POMs here. Participants reported on a scale anchored with 1 (Not at all), 3 (A little), 5 (Moderately), 7 (Quite a bit), 9 (Extremely) how anxious, on edge, and uneasy they felt in the moment that they completed the survey. Again, we computed reliability and found that the scale had an R_*c*_ of 0.77.

#### Covariates

To account for the fact that psychological stress was not measured in reference to the particular co-experienced shared stressor, we included covariates in our statistical model. The covariates are at the individual level of analysis and represent the number of other stressful events that occurred to the participant that day. Individual stressors include the 6-item checklist of stressful events used in the Midlife in the United States study: *Today*… (1) *I had an argument or disagreement with someone*, (2) *something happened that I could have argued about but I decided to let pass in order to avoid a disagreement*, (3) *something happened regarding work or school that most people would consider stressful*, (4) *something happened at home that most people would consider stressful*, (5) *something happened related to experiencing discrimination on the basis of things such as race, sex, or age*, (6) *something happened to a close friend or relative that turned out to be stressful for you*, plus a few additional items that we thought were relevant during the COVID-19 pandemic: (7) *something happened in your personal life related to coronavirus that most people would consider stressful*, (8) *something happened politically related to coronavirus and affected you, that most people would consider stressful*, (9) *something happened politically, related to racial inequalities and affect you, that most people would consider stressful*, (10) *something happened socio-politically, related to anything else (i.e*., *not coronavirus or racial inequalities) and affected you, that most people would consider stressful (i.e*., *development of an emerging socio-political crisis)*. Following the checklist items, participants were also asked to indicate the number of *other* stressful events they experienced. The sum of these items was taken and added to the number of other stressors that a person experienced based on codes from the shared stressor portion of the survey. These include stressors: (11) intrinsic to the relationship (e.g., issues in their sex life, difficult relationship conversations), (12) that did not match that of the partner and therefore did not meet the criteria for a shared stressor (e.g., person 1 said one thing happened and person 2 said another thing happened), (13) stressors that occurred to a person’s partner that they listed as stressful, but did not meet the shared stressor criteria due to the event being clearly more stressful for the partner rather than the self (e.g., a person’s partner was dealing with stressful paperwork). Participants on average reported 1.3 non-shared stressors per day (between-person *SD* = 1.0, range = 0–10).

### Statistical Model

We did visual inspections for outliers and analyzed the data using a multivariate multilevel model in the brms package of the R statistical software. There were two dependent variables: males’ and females’ self-reported anxiety, which is an operationalization of self-reported psychological stress at the end of the day. The independent variables were: (1) the 0/1 indicator of whether a co-experienced stressor occurred that day, (2) the number of other stressors experienced that day, (3) an interaction between the co-experienced stressor and participant’s reports of how much shared reality they experienced about the stressor. Note that participants could only have shared reality about a co-experienced stressor if they actually experienced a co-experienced stressor on that day. We ran the model with random intercepts and random slopes for co-experienced stressors and subjective shared reality.

Note that our use of Bayesian estimation allows us to estimate complex multilevel models that would not converge were we to use conventional frequentist, that is, Maximum Likelihood estimation (Gelman et al., 2013). Bayesian models have become increasingly user-friendly in recent years, particularly with the brms package in the R statistical software (Bürkner, 2017), available on our OSF repository at https://osf.io/t7gdf/. We hope this aids readers who wish to similarly run Bayesian estimation models on their own data.

In terms of prior values, we again choose non-informative (default) priors due to the novelty of our research question; we could not find plausible prior distributions to use. This means that our estimates are directly comparable to those that would emerge from frequentist models.

### Results

We will discuss results for males and females separately, followed by a few analyses investigating covarying effects between them. The main results can be found in Table 2.

**TABLE 2:** Multivariate regression results with shared reality predicting psychological stress.

	Females			Males		

	Average	Probability average > 0	% People > 0	Average	Probability average > 0	% People > 0
Co-experienced stressor slope	0.12	76%	96%	0.09	30%	90%
Covariate slope	0.28	>99%	NA	0.24	>99%	NA
Shared reality × co-experienced stressor slope	−0.28	5%	1%	−0.09	30%	58%

#### Females

##### The Average Female

The model revealed that the average female reported an anxiety level of 3.02. Also for the average female, the co-experienced stressor was associated with a 0.12 increase in psychological stress, and there was a 76% chance that this average effect was above zero. This reveals that co-experienced stressors increase psychological stress, holding constant shared reality and the number of other stressors. In terms of the effect of the number of other stressors on psychological stress, the average female reported a 0.28 increase for every additional stressor above her own average and the probability that this value was above zero was more than 99%. Most importantly, there was a negative relation between shared reality and psychological stress during co-experienced stressors. Each additional unit increase in shared reality above her own average corresponds to a 0.28 decrease in psychological stress below her own average. This aligns with our hypothesis that, on average, females become less stressed as shared reality about a co-experienced stressor increases. There was a 95% chance that this average slope was below zero.

##### Female-Specific Effects

Interestingly, almost every female in the sample showed effects similar to the average female, at least in terms of direction. The 96% of the females were more anxious on days that they experienced a co-experienced stressor. Unsurprisingly, more than 99% of females were more anxious on days when they experienced additional stressors and more than 99% were more stressed for each additional non-co-experienced stressor. In terms of our main effect of interest, the effect of shared reality during co-experienced stressors on reactivity, 99% of the females were less psychologically stressed as they experienced more shared reality. This robust finding suggests that the effect of shared reality on reactivity to co-experienced stressors is almost always beneficial, at least for the population of females from which our sample was obtained.

#### Males

##### The Average Male

Similar to the average female, average male reported being a 2.5 on psychological stress. The average male also experienced a slight increase in psychological stress on days with a co-experienced stressor, although the model was uncertain about this, with a probability of only 70%. In terms of other stressors, every additional stressor above the average male’s own average was associated with a 0.24 increase in psychological stress above his own average, and there was a 99% probability that this effect was greater than zero. Most importantly, we are not certain about the role of shared reality on psychological stress during a co-experienced stressor for the average male, with the model estimating a −0.1 decrease on average but there being only a 30% chance that this effect was below zero. This implies that there was no reliable effect of shared reality on psychological stress reactivity for males.

##### Male-Specific Effects

Looking at the model-predicted intercepts and slopes for each male in the sample reveals an interesting picture. More than 99% of males felt more psychological stress for each additional non-co-experienced stressor, and 90% of the males had effects greater than zero for co-experienced stressors; for almost all males, stressors increased psychological stress above their own average levels. However, the majority of males did not show a decrease in psychological stress as they shared more reality with their partners. In fact, only 42% of the males showed beneficial effects of shared reality and 58% of the males in the sample were more anxious for each additional unit increase in shared reality. This finding suggests that some (42%) of males show prototypically female effects of shared reality on stress when considered from a tend-and-befriend perspective, and that 58% of them might actually be more stressed as sharing reality increases with their romantic partners.

#### Covariances Between Females and Males

The residual correlation in the model lends insight into emotional covariation between the members of the dyad; it represents the extent to which a unit increase in partner’s average of psychological stress corresponds to an increase in the other partner’s average level of psychological stress after partialing out the independent variables in the model. Couples in this sample covaried moderately in their levels of psychological stress, with the best estimate for the correlation being 0.28 and more than 99% of the plausible values for the correlation being above 0. Because the model does not directly estimate covariances between shared reality in partners, we ran a random intercepts multivariate model with males and females shared reality as the dependent variables and the random intercepts only. This revealed the extent to which one partner’s shared reality corresponded with the other partner’s shared reality about the co-experienced stressor. The residual correlation was 0.43 and there was over a 99% probability that this correlation was larger than 0.

Finally, the primary model reveals the covariation between the effect of shared reality on psychological stress for females and the effect of shared reality on psychological stress for males. Is it true that as shared reality reduces psychological stress for a female in a dyad, it is more likely to reduce psychological stress for the man in the dyad? The estimate for the covariance was −0.07, and there was a 59% chance that this covariation was negative. We do not have evidence to conclude that the effect of shared reality on reactivity covaried between partners in the same dyad.

#### Comparing Males and Females

**FIGURE 7:**
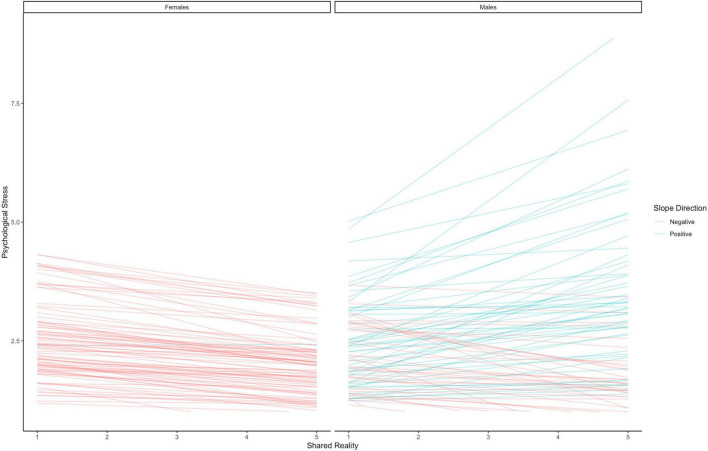
Person-specific model predicted slopes for the effect of shared reality on psychological stress.

One thing that stands out about Figure 7 is that the distribution of slopes for males seems far more heterogeneous than the distribution of slopes for females. To test this, we compared the population estimate for the standard deviation in male slopes to that for female slopes. We took the posterior distribution for the standard deviation of the effect of shared reality on psychological stress for females and subtracted the posterior distribution of the standard deviation for that slope for males (see e.g., Kruschke, 2015). Results show that the average difference between the standard deviation for females and males is −0.36 and that there is an 85% probability that this effect is indeed less than zero. In other words, males trend to being more heterogeneous in the effect of shared reality on psychological stress compared to females; but we are only 85% sure of this.

### Discussion

Results from Study 2 generalize our findings from Study 1; shared reality reduced psychological stress in everyday life, and this effect was most pronounced in females. In fact, 99% of the females in our sample showed reductions in stress on days when they experienced more shared reality in the face of a co-experienced stressor with their romantic partners. In contrast, the average male did not show beneficial effects of shared reality and 58% of the sample has slopes in the opposite direction. Nonetheless, when comparing the effect for the average female and the average male in a secondary model, we did not find statistical evidence for a reliable difference between them.

Because we find the person-specific effects to be more interesting, we will focus on those. Specifically, that 99% of females exhibited beneficial effects of shared reality while only 42% of males did.

## General Discussion

More than 20 years ago, Taylor et al. (2000) theorized that males and females respond differently to stressful situations. Males exhibit ‘fight or flight’ responses and females exhibit ‘tend-and-befriend’ responses on average. Our results deepen the field’s understanding of tend-and-befriend theory by implying a psychological process that optimizes the ability for females to tend-and-befriend: the subjective experience of shared reality. At the outset, we thought that females who create shared reality could be those who experience less psychological and physiological reactivity to co-experienced stressors. This would happen because females must swiftly bond together to ward off threats, which would be best achieved when they have a shared reality about the stressor.

We found in Study 1 that undergraduate females whose stress appraisal was validated by a confederate were those who self-reported being less psychologically stressed compared to females whose stress appraisal was either disconfirmed or left uncertain. There was robust evidence that these effects were driven by the psychological experience of shared reality, which increased epistemic certainty and relational closeness. At the physiological level, females in the shared reality condition exhibited reduced heart rate reactivity and lower levels of parasympathetic (‘rest and digest’) reduction. This latter result is especially interesting given that parasympathetic arousal is critically linked with the peptide hormone theorized to drive the tend-and-befriend response, oxytocin (Taylor et al., 2000).

Study 2 generalized these findings to non-university students, to everyday life stressors, and most importantly, to males. Here, 99% of the females in the sample were less psychologically stressed when they agreed with their romantic partner about the stressfulness of a co-experienced stressor. However, their male romantic partners typically did not show the same benefits of shared reality, although some males (42% of the sample) did.

These results provide suggestive evidence regarding interplay between sex, physiology, psychology, and behavior during stressful situations. The fact that female participants in Study 1 were less reactive when another person validated their appraisal emphasizes females’ sociality during stressful situations. Prior work has found that females are more sensitive to emotional cues in other’s faces (Hall et al., 2000; although see Fischer et al., 2018 for a caveat) and that they are better than males at mimicking other people’s facial expressions in emotion-eliciting situations (Stel and Van Knippenberg, 2008). Our results imply that these behavioral abilities also correspond with shared psychological states; female participants were highly sensitive to the shared reality manipulation in Study 1. Such sensitivity altered downstream reactivity to the stressor, which implies that females’ capacity to understand and align with each other’s emotions has real consequences for physiology and health. The fact that Study 1 found reductions in parasympathetic withdrawal implies that creating the shared reality clamed participants down, rather than the other conditions amplifying reactivity. To test the causal relation between shared reality and stressor reactivity at the physiological and psychological levels would require a replication and extension of this study that includes male-male interactions during a co-experienced stressor. We await future studies to collect and analyze such data.

We find our preliminary results interesting in light of the fact that most stressful situations in the modern world include other people; people must jointly tackle stressful work situations with a boss, colleague, or subordinate, people must jointly deal with family issues in a complicated healthcare landscape, and people mutually face situations like delayed transportation and climate change with strangers. Fighting or fleeing is unlikely to help people deal with these types of stressors. Our work implies that females may be especially adept at dealing with the types of stressors they face, at least in the United States, as they benefit from the psychological experience of aligning with another person on the meaning of those stressful situations. The fact that 99% of females were less psychologically stressed when experiencing shared reality with their partners in Study 2 shows how widespread these benefits might be.

Moreover, our results from Study 2 highlight our inability to talk about these effects as being perfectly moderated by sex. Taylor et al. (2000) asserted that tend-and-befriend theory concerns average effects for males and females, and that those biologically driven tendencies would not map perfectly onto every person’s reactivity to stressful situations. Culture and role theories of gender also imply that males and females could vary in their tendency to follow norms; i.e., that even females who generally fulfill normative roles could sway from female typicality in certain situations. Our study leveraged the power of intensive longitudinal data to test and better understand these claims. While we found compelling evidence that the result is more consistent among females, there was *not* a 1–1 correspondence between sex and the effects of shared reality on stressor reactivity. Instead, most females and some males exhibited beneficial effects of shared reality while the remaining men showed harmful effects. This raises the question: If shared reality is a psychological process that enables females to tend-and-befriend effectively, then why do some males benefit from shared reality during co-experienced stressors?

One answer could be that some males are more biologically similar to females. From an evolutionary perspective, it could be that male tending and befriending was adaptive for some males. Prior research has found male-male and female-male bondedness among non-human primates under conditions of low external threat (i.e., resources, predators, etc.; Van Schaik and Van Hooff, 1994). Biologically then, some males may have adapted to bond with others so that in the rare instances that they were exposed to threat, they could make use of the social bonds that benefit them in daily life. If this were true, then some males might exhibit the same uptick in oxytocin during stressors. While no known research has examined the distribution of oxytocin levels among males during stressors, we do know that males who receive endogenous administration of oxytocin during stressful situations exhibit swifter vagal rebound (Kubzansky et al., 2012), which is linked to tending and befriending.

Another potential answer is that some males may have been socialized in ways that override their biological propensity to fight or flee during threat. We know that gender roles relate to emotional expression; males and females who have stronger stereotypes about gender and emotion are those who show greatest differences in emotion expression (Grossman and Wood, 1993). The corollary is that males who are less gender-stereotypic, expressing their emotions truthfully and therefore aligning with others during co-experienced stressors, may reap the benefits of tending and befriending in response to co-experienced stressors. Indeed, theories about how and why people share emotions with others in the aftermath of traumatic events make no mention of sex or gender (see Harber et al., 2014; Harber and Cohen, 2016), likely because the proposed psychological benefits of doing so transcend biological and role differences between males and females. Because we only studied biological males and females whose gender identities align with their sex assigned at birth, we are unable to empirically differentiate whether our results are driven by biology or gender. We hope future researchers will collect data on the effects of shared reality on stress among people whose gender does not align with their sex assigned at birth in order to unconfound sex from gender, as well better represent the gender spectrum that we know to be non-binary (Vijlbrief et al., 2020).

At first glance our results seem contradictory to existing findings in the emotion literature. Specifically, existing research on co-experienced events finds amplification rather than dampening (Boothby et al., 2014). In these studies, most shared experiences are thought to amplify sensory and psychological outcomes because the other person increases the vividness of the experience. This was reflected in Study 2, as 58% of the males were more psychologically stressed as shared reality increased with their partners. In further support of the amplification hypothesis, research on emotion contagion implies negative effects of social sharing during stressful times. Mothers who are exposed to a lab-based stressor transfer their stress to their infant, presumably because the infant is attuned to how the mother feels (Waters et al., 2014). Finally, in adult dyads physiological covariation can lead to increased stress, as higher levels of being physiologically ‘in tune’ can lead romantic partners to “catch” each other’s heightened arousal (Levenson and Gottman, 1983; Chen et al., 2020). This occurs because being overly connected during negative interpersonal interactions leads to a feedback system that amplifies negative emotionality (Butler, 2011). Why, then, did we observe reduced reactivity and beneficial outcomes for most females and some males in the kind of social sharing we studied: shared reality?

We think that one answer is that prior work has rarely considered the psychological process that occurs during co-experienced events. Work by Boothby and colleagues has, to date, only investigated reactivity among participants who are either alone or merely present with another person. With this design, it is possible that reactivity within the ‘shared’ condition is itself moderated by the psychological experience of shared reality. Future research could compare being alone to being with another person *and* having shared reality to being with another person but *not* having shared reality. Moreover, studies in the amplification literature rarely provide opportunities for shared reality to be created. For example, one study compared emotional reactivity to computer-based stimuli when there was “another online participant” versus not (Shteynberg et al., 2014). With such a design, participants might not create any psychological connection with the conspecific because that other person was only present online. Indeed, experience amplification depends on psychological proximity, operationalized as being in the same room versus in a separate room and being with someone acquainted to the participant versus being with a complete stranger (Boothby et al., 2016). We therefore think our findings might complement the amplification literature, as amplifying effects might only occur when shared reality is not created and only for some people.

Other research that points to amplification during co-experiences, specifically physiological amplification, is also different from our work in that it investigates endogenous stressors. In the physiological covariation literature, participants discuss problems that occur between them rather than experience stressors that are external to their relationships. While agreeing about the stressfulness of an ongoing endogenous stressors can lead to a psychological spiral toward negativity, shared reality during exogenous stressors need not do so. Altogether, our findings suggest that it is *not* the case that social sharing always amplifies experiences, that emotional transmission is always bad, or that linkage always exerts negative effects. Rather, subtle differences in psychological processes during a stressor, who is implicated in it, and what is shared, matters.

We need to note some limitations in our research that constrain the generalizability and interpretability of our results. Importantly, we did not pre-register our research and our results should be considered preliminary. Next, Study 1 contained a sample of only undergraduate female students and Study 2 contained a sample of only heteronormative couples in the New York City area. To extrapolate our findings to people who are not represented by the populations from which we sampled would be inappropriate, and future research should focus on generalizability now that we have presented preliminary findings. With our data we are unable to fully differentiate biological tend-and-befriend differences from role differences emerging from cultural expectations of males and females. To fully disconfound sex and gender requires sampling from populations in which sex assigned at birth and gender do not align (i.e., the trans community) or more careful sampling of biological mechanisms, specifically oxytocin. We generated our hypotheses and interpreted our results in light of tend-and-befriend theory and role theories of gender because we find the hypotheses generated from those literatures to align with our results. Nonetheless, we encourage future researchers to empirically disaggregate these overlapping causal explanations. Another limitation is our focus on only one co-experienced stressor. In Study 1, participants underwent a single stressful task and in Study 2, we only asked participants about one co-experienced stressor each day. It is possible that compounded co-experienced stressors, and the amount of shared reality experienced for each of them, could have unique and multiplicative effects. Finally, we only considered shared reality during co-experienced stressors for stressors that are relatively mild. It could be possible that curvilinear effects emerge for shared reality and stressor reactivity as the intensity of the stressor grows; i.e., perhaps shared reality during highly threatening situations leads to maladaptive outcomes because having one’s appraisal validated for an extreme stressor could lead to the freeze response (see Bracha, 2004). Nonetheless, our research lays the foundation for future research on shared reality and stressor reactivity that could address each of these limitations.

Before concluding, we would also like to briefly discuss potential boundary conditions of these effects. One of our reviewers inquired about the moderating role of attributions, specifically that stressors that elicit reactivity due to internal attributions are likely not susceptible to shared reality. For example, reactivity to an upcoming exam could be explained by the amount of time a student spent studying insofar as study time is used as an attribution for feelings of anxiousness and nervousness. However, existing scholarship emphasizes the multi-faceted nature of attributions during the stress process. Time spent studying for an exam could lead a student to be stress reactive, but when they see that other students are also anxious about the exam they might believe that innate ability will contribute to performance and therefore feel less psychologically stressed. Future research could empirically test this quandaries by investigating how attributions for stress moderate the effect of shared reality on reactivity.

## Conclusion

In this study, we leveraged shared reality theory to posit that agreeing with someone about the stressfulness of a co-experienced stressor could reduce reactivity for females by enabling them to efficiently tend-and-befriend with other females. Results from two preliminary studies support our hypotheses, pointing to the beneficial effect of shared reality during co-experienced stressors on reactivity for females and even for some males. We highlighted the interactive nature of psychological, physiological, and social processes that underlie these effects. Together, this work implies that shared reality plays a critical role in stressor reactivity among females and some males. We invite future researchers to deepen the theoretical implications of these findings as the field continues to investigate the role of shared reality, biology, and sex in reactivity to co-experienced stressors.

## Data Availability Statement

The datasets presented in this study can be found in online repositories. The repository can be found here: https://osf.io/t7gdf/.

## Ethics Statement

The studies involving human participants were reviewed and approved by Columbia University Institutional Review Board. The participants provided written informed consent to participate in this study.

## Author Contributions

MG had the initial idea to test whether shared reality reduces or amplifies reactivity to stressful events. She conducted the primary literature review on the topic and recruited FP to help brainstorm the research ideas, provide insight on study design, and contribute expertise on shared reality theory. All work was supervised by NB and ETH. Study 1 was co-designed by MG and FP. They both recruited and trained research assistants on how to run participants through the study. MG managed all aspects of data collection and data cleaning. She also analyzed and visualized the data. In Study 2, MG designed the study, trained research assistants on how to run participants through the protocol, and cleaned the data with the help of a research assistant. FP advised on measurement in Study 2. The manuscript was written by MG and edited by FP, NB, and ETH. NB and ETH funded this research. All authors contributed to the article and approved the submitted version.

## Conflict of Interest

The authors declare that the research was conducted in the absence of any commercial or financial relationships that could be construed as a potential conflict of interest.

## Publisher’s Note

All claims expressed in this article are solely those of the authors and do not necessarily represent those of their affiliated organizations, or those of the publisher, the editors and the reviewers. Any product that may be evaluated in this article, or claim that may be made by its manufacturer, is not guaranteed or endorsed by the publisher.

## Footnotes

1 Because we are focused on sex assigned at birth, we use the terms male and female throughout the manuscript.

2
https://osf.io/t7gdf/

3 Note that due to chance, our randomization procedure did not equally distribute participants into the three conditions in terms of number or appraisal score; participants in the SRD condition appraised the speech as more stressful than those in the SRC and AMB conditions. To account for this, we ran our models controlling for appraisal and the results remain the same. See our OSF materials (https://osf.io/t7gdf/) to run this model and view the results of this analysis.

4 We also used a third item, *I have a pretty good idea of what giving the speech entails*, that lowered the Cronbach’s alpha considerably, to 0.56. When running the model with that item included, the results are almost identical.

## References

[B1] Allen K. M. Blascovich J. Tomaka J. Kelsey R. M. (1991). Presence of human friends and pet dogs as moderators of autonomic responses to stress in females. *J. Personal. Soc. Psychol.* 61 582–589. 10.1037/0022-3514.61.4.582 1960650

[B2] Almeida D. M. (2005). Resilience and vulnerability to daily stressors assessed via diary methods. *Curr. Direct. Psychol. Sci.* 14 64–68. 10.1111/j.0963-7214.2005.00336.x

[B3] Almeida D. M. Wethington E. Kessler R. C. (2002). The daily inventory of stressful events: An interview-based approach for measuring daily stressors. *Assessmalest* 9 41–55. 10.1177/1073191102091006 11911234

[B4] Appelbaum M. Cooper H. Kline R. B. Mayo-Wilson E. Nezu A. M. Rao S. M. (2018). Journal article reporting standards for quantitative research in psychology: The APA Publications and Communications Board task force report. *Am. Psychol.* 73 3–25. 10.1037/amp0000191 29345484

[B5] Bellingtier J. A. Neupert S. D. Kotter-Grühn D. (2017). The combined effects of daily stressors and major life events on daily subjective ages. *J. Gerontol. Ser. B* 72 613–621. 10.1093/geronb/gbv101 26582213

[B6] Berg C. A. Wiebe D. J. Butner J. (2011). Affect covariation in marital couples dealing with stressors surrounding prostate cancer. *Gerontology* 57 167–172. 10.1159/000318642 20616529

[B7] Blascovich J. Vanman E. Malesdes W. B. Dickerson S. (2011). *Social Psychophysiology for Social and Personality Psychology.* Thousand Oaks: Sage Publications.

[B8] Bolger N. Laurenceau J. P. (2013). *Intensive Longitudinal Methods: An Introduction to Diary and Experience Sampling Research.* New York, NY: Guilford Press.

[B9] Boothby E. J. Clark M. S. Bargh J. A. (2014). Shared experiences are amplified. *Psychol. Sci.* 25 2209–2216. 10.1177/0956797614551162 25274583

[B10] Boothby E. J. Smith L. K. Clark M. S. Bargh J. A. (2016). Psychological distance moderates the amplification of shared experience. *Personal. Soc. Psychol. Bull.* 42 1431–1444. 10.1177/0146167216662869 27562770

[B11] Bracha H. S. (2004). Freeze, flight, fight, fright, faint: Adaptationist perspectives on the acute stress response spectrum. *CNS Spectr.* 9 679–685. 10.1017/S1092852900001954 15337864

[B12] Brody L. R. (1985). Gender differences in emotional development: A review of theories and research. *J. Personal.* 53 102–149. 10.1111/j.1467-6494.1985.tb00361.x

[B13] Bürkner P. C. (2017). brms: An R Package for Bayesian Multilevel Models Using Stan. *J. Statist. Softw.* 80 1–28. 10.18637/jss.v080.i01

[B14] Butler E. A. (2011). Temporal interpersonal emotion systems: The “TIES” that form relationships. *Personal. Soc. Psychol. Rev.* 15 367–393. 10.1177/1088868311411164 21693670

[B15] Camerino C. (2009). Low sympathetic tone and obese phenotype in oxytocin-deficient mice. *Obesity* 17 980–984. 10.1016/j.psyneuen.2011.05.007 19247273

[B16] Chen K. H. Brown C. L. Wells J. L. Rothwell E. S. Otero M. C. Levenson R. W. (2020). Physiological linkage during shared positive and shared negative emotion. *J. Personal. Soc. Psychol.* 121 1029–1056. 10.1037/pspi0000337 32897091 PMC8261768

[B17] Costa-e-Sousa R. H. Pereira-Junior P. P. Oliveira P. F. Olivares E. L. Werneck-de-Castro J. P. S. Mello D. B. (2005). Cardiac effects of oxytocin: is there a role for this peptide in cardiovascular homeostasis? *Regulat. Pept.* 132 107–112. 10.1016/j.regpep.2005.09.011 16213606

[B18] Cottrell N. B. Epley S. W. (1977). “Affiliation, social comparison and socially mediated stress reduction,” in *Social Comparison Processes: Theoretical and Empirical Perspectives*, eds Suls J. M. Miller R. L. (New York, NY: Hemisphere), 43–68. 10.1186/s12888-018-1863-z

[B19] Cuff B. M. Brown S. J. Taylor L. Howat D. J. (2016). Empathy: A review of the concept. *Emot. Rev.* 8 144–153. 10.1177/1754073914558466

[B20] Diamond L. M. Aspinwall L. G. (2003). Emotion regulation across the life span: An integrative perspective emphasizing self-regulation, positive affect, and dyadic processes. *Motivat. Emot.* 27 125–156. 10.1023/A:1024521920068

[B21] Dienes Z. (2011). Bayesian versus orthodox statistics: Which side are you on? *Perspect. Psychol. Sci.* 6 274–290. 10.1177/1745691611406920 26168518

[B22] Echterhoff G. Higgins E. T. Groll S. (2005). Audience-tuning effects on memory: the role of shared reality. *J. Personal. Soc. Psychol.* 89 257–276. 10.1037/0022-3514.89.3.257 16248713

[B23] Echterhoff G. Higgins E. T. Levine J. M. (2009). Shared reality: Experiencing commonality with others’ inner states about the world. *Perspect. Psychol. Sci.* 4 496–521. 10.1111/j.1745-6924.2009.01161.x 26162223

[B24] Echterhoff G. Kopietz R. Higgins E. T. (2013). Adjusting shared reality: Communicators’ memory changes as their connection with their audience changes. *Soc. Cogn.* 31 162–186. 10.1521/soco.2013.31.2.162

[B25] Epel E. S. Crosswell A. D. Mayer S. E. Prather A. A. Slavich G. M. Puterman E. (2018). More than a feeling: A unified view of stress measurement for population science. *Front. Neuroendocrinol.* 49:146–169. 10.1016/j.yfrne.2018.03.001 29551356 PMC6345505

[B26] Feldman R. (2012). Oxytocin and social affiliation in humans. *Hormones Behav.* 61 380–391. 10.1016/j.yhbeh.2012.01.008 22285934

[B27] Felnhofer A. Kaufmann M. Atteneder K. Kafka J. X. Hlavacs H. Beutl L. (2019). The mere presence of an attentive and emotionally responsive virtual character influences focus of attention and perceived stress. *Int. J. Hum. Comput. Stud.* 132 45–51. 10.1016/j.ijhcs.2019.07.010

[B28] Festinger’s social comparison theory (1957). *A Theory of Cognitive Dissonance.* Redwood City: Stanford University Press.

[B29] Fischer A. H. Kret M. E. Broekens J. (2018). Gender differences in emotion perception and self-reported emotional intelligence: A test of the emotion sensitivity hypothesis. *PLoS One* 13:e0190712. 10.1371/journal.pone.0190712 29370198 PMC5784910

[B30] Frenzel A. C. Becker-Kurz B. Pekrun R. Goetz T. Lüdtke O. (2018). Emotion transmission in the classroom revisited: a reciprocal effects model of teacher and student enjoymalest. *J. Educat. Psychol.* 110 628–639. 10.1037/edu0000228

[B31] Gelman A. Carlin J. B. Stern H. S. Dunson D. B. Vehtari A. Rubin D. B. (2013). *Bayesian Data Analysis.* London: CRC Press.

[B32] Goldring M. R. Bolger N. (2021). Physical Effects of Daily Stressors Are Psychologically Mediated, Heterogeneous, and Bidirectional. *J. Personal. Soc. Psychol.* 121 722–746. 10.1037/pspp0000396 34807700

[B33] Goodman W. K. Janson J. Wolf J. M. (2017). Meta-analytical assessmalest of the effects of protocol variations on cortisol responses to the Trier Social Stress Test. *Psychoneuroendocrinology* 80 26–35. 10.1016/j.psyneuen.2017.02.030 28292684

[B34] Grossman M. Wood W. (1993). Sex differences in intensity of emotional experience: a social role interpretation. *J. Personal. Soc. Psychol.* 65 1010–1022. 10.1037/0022-3514.65.5.1010 8246109

[B35] Hall J. A. Carter J. D. Horgan T. G. Fischer A. H. (eds) (2000). “Gender differences in nonverbal communication of emotion,” in *Gender and Emotion: Social Psychological Perspectives*, (Cambridge: Cambridge University Press), 97–117. 10.1017/CBO9780511628191.006

[B36] Harber K. D. Cohen D. J. (2016). The emotional broadcaster theory of social sharing. *J. Lang. Soc. Psychol.* 24 382–400. 10.1177/0261927x05281426

[B37] Harber K. D. Podolski P. Dyer L. (2014). Hearing stories that violate expectations leads to emotional broadcasting. *J. Lang. Soc. Psychol.* 33 5–28. 10.1177/0261927X13502793

[B38] Hardin C. D. Higgins E. T. (1996). “Shared reality: How social verification makes the subjective objective,” in *Handbook of Motivation and Cognition the Interpersonal Context*, eds Sorrentino R. M. Higgins E. T. (New York, NY: The Guilford Press), 28–84.

[B39] Higgins E. T. (2019). *Shared Reality: What Makes Us Strong and Tears us Apart.* Oxford, UK: Oxford University Press.

[B40] Jezova D. Jurankova E. Mosnarova A. Kriska M. Skultetyova I. (1996). Neuroendocrine response during stress with relation to gender differences. *Acta Neurobiol. Exp.* 56 779–785. 8917906 10.55782/ane-1996-1183

[B41] Joo S. Chai H. W. Jun H. J. Almeida D. M. (2020). Daily stressors facilitate giving and receiving of emotional support in adulthood. *Stress Health* 36 330–337. 10.1002/smi.2927 31957983 PMC7369222

[B42] Kirschbaum C. Pirke K. M. Hellhammer D. H. (1993). The ‘Trier Social Stress Test’–a tool for investigating psychobiological stress responses in a laboratory setting. *Neuropsychobiology* 28 76–81. 10.1159/000119004 8255414

[B43] Kotter-Grühn D. Neupert S. D. Stephan Y. (2015). Feeling old today? Daily health, stressors, and affect explain day-to-day variability in subjective age. *Psychol. Health* 30 1470–1485. 10.1080/08870446.2015.1061130 26066614

[B44] Kruschke J. (2015). *Doing Bayesian Data Analysis: A Tutorial with R, JAGS, and Stan*, 2nd Edn. Cambridge, UK: Academic Press.

[B45] Kubzansky L. D. Malesdes W. B. Appleton A. A. Block J. Adler G. K. (2012). A heartfelt response: oxytocin effects on response to social stress in males and females. *Biol. Psychol.* 90 1–9. 10.1016/j.biopsycho.2012.02.010 22387929 PMC3327158

[B46] Kulik J. A. Mahler H. I. M. (2000). “Social Comparison, Affiliation, and Emotional Contagion under Threat,” in *Handbook of Social Comparison. The Springer Series in Social Clinical Psychology*, eds Suls J. Wheeler L. (Boston, MA: Springer), 10.1007/978-1-4615-4237-7_15

[B47] Lazarus R. S. (1993). From psychological stress to the emotions: A history of changing outlooks. *Annu. Rev. Psychol.* 44 1–22. 10.1146/annurev.ps.44.020193.000245 8434890

[B48] Levenson R. W. Gottman J. M. (1983). Marital interaction: physiological linkage and affective exchange. *J. Personal. Soc. Psychol.* 45:587. 10.1037/0022-3514.45.3.587 6620126

[B49] Mauss I. B. Robinson M. D. (2009). Measures of emotion: A review. *Cogn. Emot.* 23 209–237. 10.1080/02699930802204677 19809584 PMC2756702

[B50] McNair P. M. Lorr M. Droppleman L. F. (1981). *Manual for the Profile of Mood States.* San Diego: EdITS Educational and Industrial Testing Service.

[B51] Mendes W. B. Blascovich J. Major B. Seery M. (2001). Challenge and threat responses during downward and upward social comparisons. *Europ. J. Soc. Psychol.* 31 477–497. 10.1002/ejsp.80

[B52] Moberg K. U. Handlin L. Kendall-Tackett K. Petersson M. (2019). Oxytocin is a principal hormone that exerts part of its effects by active fragments. *Med. Hypothes.* 133:109394. 10.1016/j.mehy.2019.109394 31525634

[B53] Nickels N. Kubicki K. Maestripieri D. (2017). Sex differences in the effects of psychosocial stress on cooperative and prosocial behavior: evidence for ‘flight or fight’in males and ‘tend-and-befriend’in females. *Adapt. Hum. Behav. Physiol.* 3 171–183. 10.1007/s40750-017-0062-3

[B54] Ochsner K. N. (2019). “From the Self to the Social Regulation of Emotion: An Evolving Psychological and Neural Model,” in *Emotion in the Mind and Body*, eds Neta M. Haas I. (Cham: Springer), 43–75.

[B55] Petersson M. Alster P. Lundeberg T. Uvnas-Moberg K. (1996). Oxytocin causes a long-term decrease of blood pressure in female and male rats. *Physiol. Behav.* 60 1311–1315. 10.1016/S0031-9384(96)00261-2 8916187

[B56] Prochazkova E. Kret M. E. (2017). Connecting minds and sharing emotions through mimicry: A neurocognitive model of emotional contagion. *Neurosci. Biobehav. Rev.* 80 99–114. 10.1016/j.neubiorev.2017.05.013 28506927

[B57] Qi Y. Herrmann M. J. Bell L. Fackler A. Han S. Deckert J. (2020). The mere physical presence of another person reduces human autonomic responses to aversive sounds. *Proc. Roy. Soc. B* 287:1919. 10.1098/rspb.2019.2241 31964306 PMC7015327

[B58] Randall A. K. Bodenmann G. (2017). Stress and its associations with relationship satisfaction. *Curr. Opin. Psychol.* 13 96–106. 10.1016/j.copsyc.2016.05.010 28813303

[B59] Rimé B. (2009). Emotion elicits the social sharing of emotion: Theory and empirical review. *Emot. Rev.* 1 60–85. 10.1177/1754073908097189

[B60] Rossignac-Milon M. Bolger N. Zee K. S. Boothby E. J. Higgins E. T. (2021). Merged minds: Generalized shared reality in dyadic relationships. *J. Personal. Soc. Psychol.* 120 882–911. 10.1037/pspi0000266 32673045

[B61] Rossignac-Milon M. Higgins E. T. (2018b). Epistemic companions: Shared reality developmalest in close relationships. *Curr. Opin. Psychol.* 23 66–71. 10.1016/j.copsyc.2018.01.001 29360060

[B62] Rossignac-Milon M. Higgins E. T. (2018a). Beyond intrapersonal cognitive consistency: Shared reality and the interpersonal motivation for truth. *Psychol. Inq.* 29 86–93. 10.1080/1047840X.2018.1480688

[B63] Ruffman T. Lorimer B. Scarf D. (2017). Do infants really experience emotional contagion? *Child Devel. Perspect.* 11 270–274. 10.1111/cdep.12244

[B64] Ryan R. M. (1982). Control and information in the intrapersonal sphere: An extension of cognitive evaluation theory. *J. Personal. Soc. Psychol.* 43 450–461. 10.1037/0022-3514.43.3.450

[B65] Schachter S. (1959). *The Psychology of Affiliation.* Stanford, CA: Stanford University Press.

[B66] Sherwood A. Allen M. T. Fahrenberg J. Kelsey R. M. Lovallo W. R. van Doornen L. J. (1990). Methodological guidelines for impedance cardiography. *Psychophysiology* 27, 1–23. 10.1111/j.1469-8986.1990.tb02171.x 2187214

[B67] Shteynberg G. Hirsh J. B. Apfelbaum E. P. Larsen J. T. Galinsky A. D. Roese N. J. (2014). Feeling more together: Group attention intensifies emotion. *Emotion* 14 1102–1114. 10.1037/a0037697 25151520

[B68] Steel C. Macdonald J. Schröder T. Mellor-Clark J. (2015). Exhausted but not cynical: Burnout in therapists working within improving access to psychological therapy services. *J. Mental Health* 24 33–37. 10.3109/09638237.2014.971145 25587817

[B69] Stel M. Van Knippenberg A. (2008). The role of facial mimicry in the recognition of affect. *Psychol. Sci.* 19 984–985. 10.1111/j.1467-9280.2008.02188.x 19000207

[B70] Taylor S. (2011). “Social psychology: A review,” in *The Oxford Handbook of Health Psychology (1st ed)*, ed. Friedman H. S. (Oxford, UK: Oxford University Press), 10.1093/oxfordhb/9780195342819.013.0009

[B71] Taylor S. E. (2006). Tend-and-befriend: Biobehavioral bases of affiliation under stress. *Curr. Direct. Psychol. Sci.* 15 273–277. 10.1111/j.1467-8721.2006.00451.x

[B72] Taylor S. E. Klein L. C. Lewis B. P. Gruenewald T. L. Gurung R. A. Updegraff J. A. (2000). Biobehavioral responses to stress in females: tend-and-befriend, not fight-or-flight. *Psychol. Rev.* 107 411–429. 10.1037/0033-295X.107.3.411 10941275

[B73] Taylor S. E. Master S. L. (2011). *Social Responses to Stress: the Tend-and-Befriend Model. the Handbook of Stress Science: Biology, Psychology, and Health (1st ed).* New York, NY: Springer Publishing Company.

[B74] Timmons A. C. Margolin G. Saxbe D. E. (2015). Physiological linkage in couples and its implications for individual and interpersonal functioning: A literature review. *J. Fam. Psychol.* 29 720–731. 10.1037/fam0000115 26147932 PMC4593729

[B75] Uvnäs-Moberg K. (1998). Oxytocin may mediate the benefits of positive social interaction and emotions. *Psychoneuroendocrinology* 23 819–835. 10.1016/S0306-4530(98)00056-0 9924739

[B76] Van De Schoot R. Winter S. D. Ryan O. Zondervan-Zwijnenburg M. Depaoli S. (2017). A systematic review of Bayesian articles in psychology: The last 25 years. *Psychol. Methods* 22 217–239. 10.1037/met0000100 28594224

[B77] Van Schaik C. P. Van Hooff J. A. (1994). Male bonds: afilliative relationships among nonhuman primate males. *Behaviour* 130 309–337. 10.1163/156853994X00587

[B78] Vijlbrief A. Saharso S. Ghorashi H. (2020). Transcending the gender binary: Gender non-binary young adults in Amsterdam. *J. LGBT Youth* 17 89–106. 10.1080/19361653.2019.1660295

[B79] Wagenmakers E. J. (2007). A practical solution to the pervasive problems of p values. *Psychon. Bull. Rev.* 14 779–804. 10.3758/BF03194105 18087943

[B80] Waters S. F. West T. V. Malesdes W. B. (2014). Stress contagion: Physiological covariation between mothers and infants. *Psychol. Sci.* 25 934–942. 10.1177/0956797613518352 24482403 PMC4073671

[B81] Wild B. Erb M. Bartels M. (2001). Are emotions contagious? Evoked emotions while viewing emotionally expressive faces: quality, quantity, time course and gender differences. *Psych. Res.* 102 109–124. 10.1016/S0165-1781(01)00225-6 11408051

[B82] Zaki J. (2020). Integrating empathy and interpersonal emotion regulation. *Annu. Rev. Psychol.* 71 517–540. 10.1146/annurev-psych-010419-050830 31553672

[B83] Zaki J. Williams W. C. (2013). Interpersonal emotion regulation. *Emotion* 13 803–810. 10.1037/a0033839 24098929

[B84] Zee K. S. Bolger N. Higgins E. T. (2020). Regulatory effectiveness of social support. *J. Personal. Soc. Psychol.* 119 1316–1358. 10.1037/pspi0000235 32052988

