# Supplementary material for: Shared Reality Can Reduce Stressor Reactivity
Source: Front Psychol. 2022 Apr 27;13:853750. doi: 10.3389/fpsyg.2022.853750 (PMC9093073; doi:10.3389/fpsyg.2022.853750)

# Supplemental Materials

## Study 1

### Deattenuated Correlation Matrix

The table below shows the deattenuated correlation matrix. Values on the diagonal are the reliabilities for each measure, values below the diagonal are raw correlations, and values above the diagonal are diattenuated correlations.

| | Target Shared Reality | Epistemic Motives Fulfilled | Relational Motives Fulfilled | Psychological Stress |
|---|---|---|---|---|
| Target Shared Reality | .94 | .28 | .39 | -.36 |
| Epistemic Motives Fulfilled | .23 | .76 | .19 | -.35 |
| Relational Motives Fulfilled | .34 | .15 | .80 | -.18 |
| Psychological Stress | -.32 | -.28 | -.14 | .83 |

### Manipulation Deep Dive

As seen in the figure below, almost all of the participants in our sample appraised the speech task as a 3 (rounded) or higher. This meant that participants in the shared reality disconfirmed condition were paired with a confederate who were less stressed than them, although there was sufficient representation below the midpoint of the scale such that some participants were paired with a confederate who appraised the speech as more stressful than them. The two participants who were a 2 or lower on the appraisal questionnaire were included in the analysis so as to avoid bias, even though at first glance it seems that they could be excluded since they did not view the speech as stressful (see Aranow et. al, 2019, doi: 10.1017/pan.2019). However, the Trier Social Stress Task is known to elicit cortisol and physiological reactivity, even for people who do not self-report feeling stressed (see Maus et. al, 2015, doi: 10.1037/1528-3542.5.2.175). Because we measure physiological reactivity, it is especially important to include those participants.

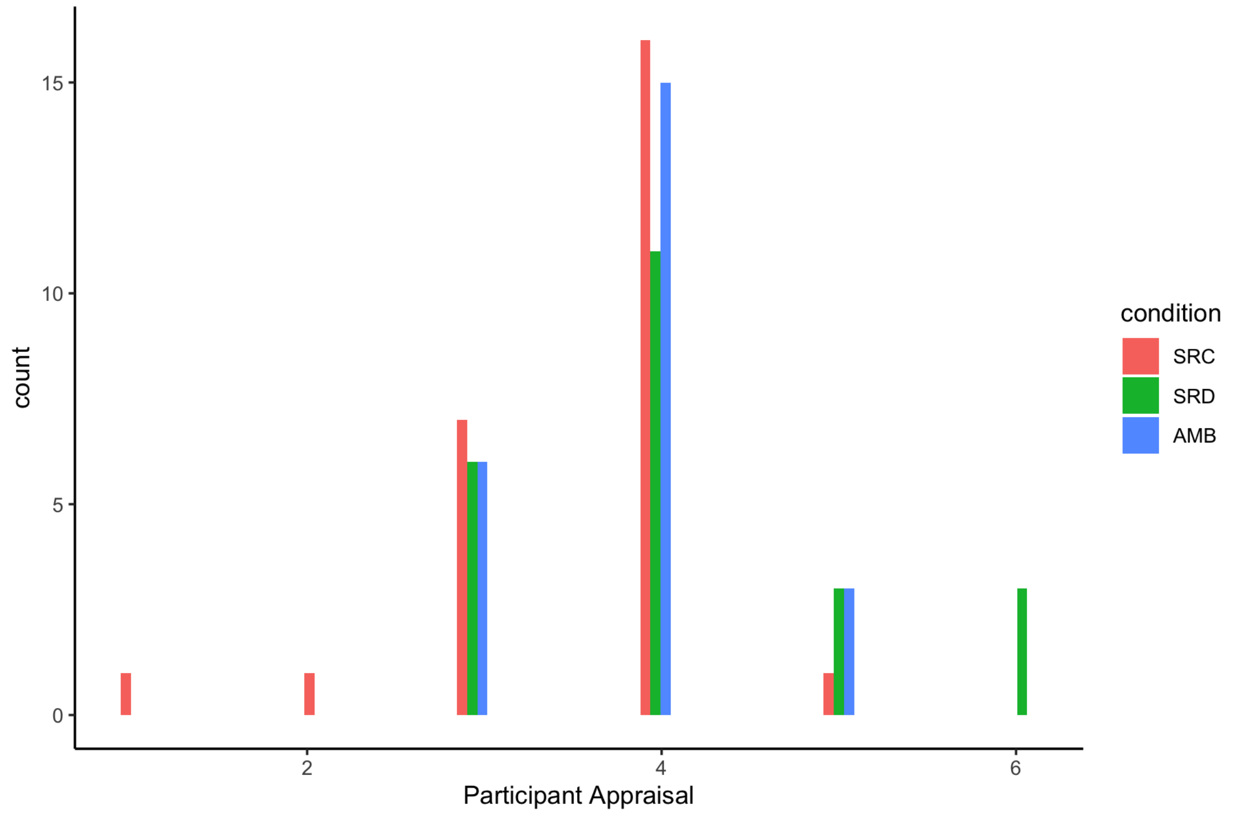


There is a potential problem that participants in the SRD condition were paired with a confederate who viewed the speech as more stressful than they did, while other participants were paired with a confederate who viewed the speech as less stressful than they did, we conducted a secondary analysis. In this analysis, we regressed psychological stress on condition controlling for initial appraisal. By doing this, we partial out any effects in the shared reality disconfirmed condition whereby the manipulation exerted a different effect depending on whether the confederate appraised the stressor as more or less stressful than they did.

| | **Model Predicted Mean Difference** | **Finding** | **Probability** |
|---|---|---|---|
| **SRC < SRD** | 0.6 | Yes | 99% |
| **SRC < SRD** | 0.3 | Yes | 91% |

Raw Data Visualizations (error bars are 95% CIs)

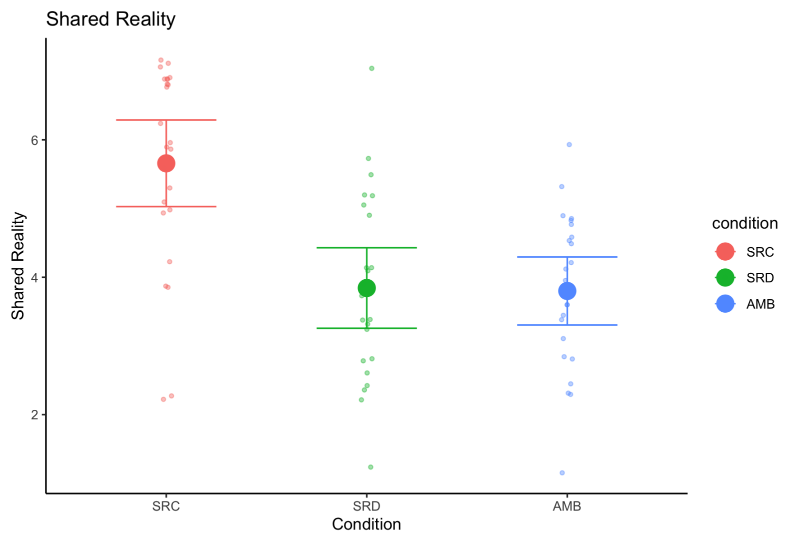

Shared Reality
Shared Reality
6
4
2
SRC
SRD
AMB
Condition
condition
SRC
SRD
AMB


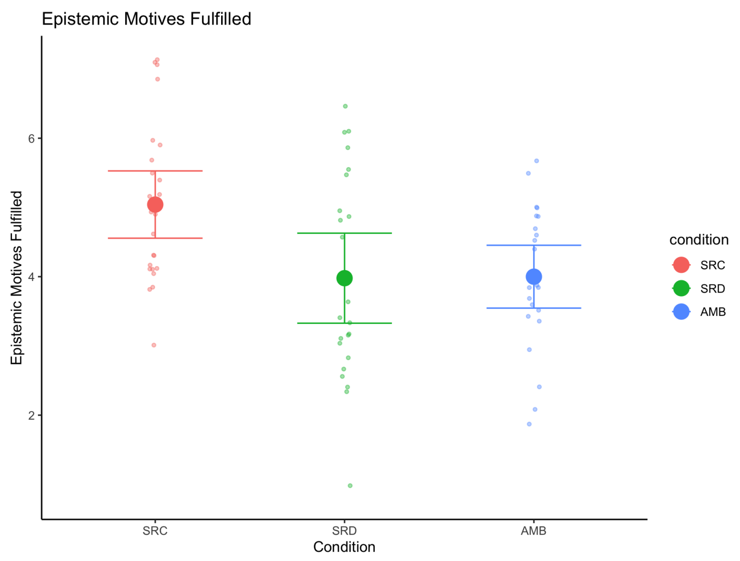

Epistemic Motives Fulfilled
Epistemic Motives Fulfilled
6
4
2
SRC
SRD
AMB
Condition
condition
SRC
SRD
AMB

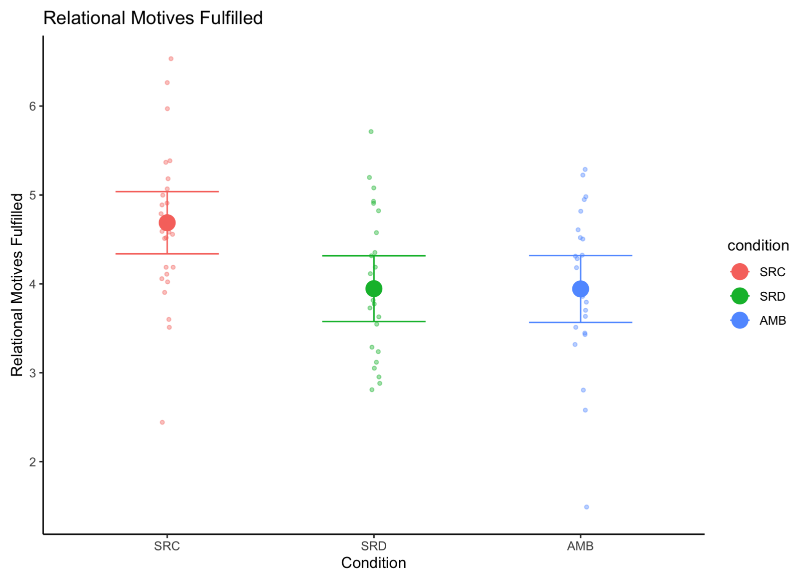

Relational Motives Fulfilled
6
5
4
3
2
Relational Motives Fulfilled
SRC
SRD
AMB
Condition
condition
SRC
SRD
AMB


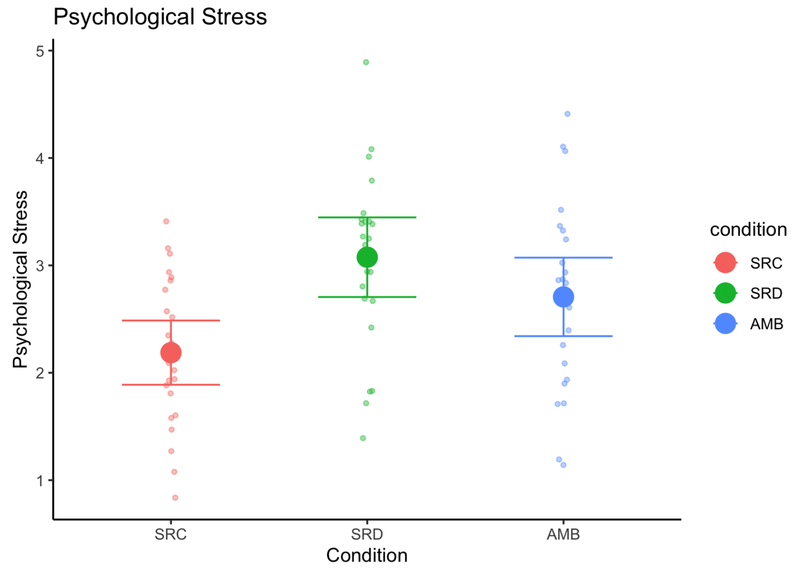

Psychological Stress
5
4
3
2
1
Psychological Stress
SRC
SRD
AMB
Condition
condition
SRC
SRD
AMB

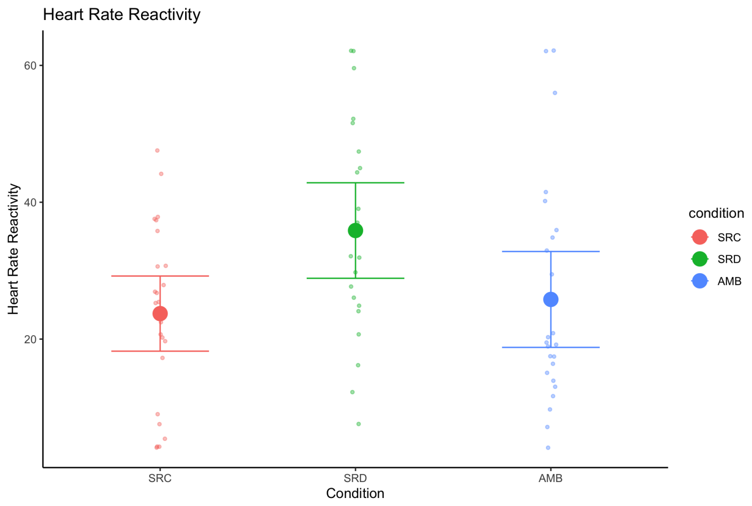
Heart Rate Reactivity
Heart Rate Reactivity
60
40
20
SRC
SRD
AMB
Condition
condition
SRC
SRD
AMB

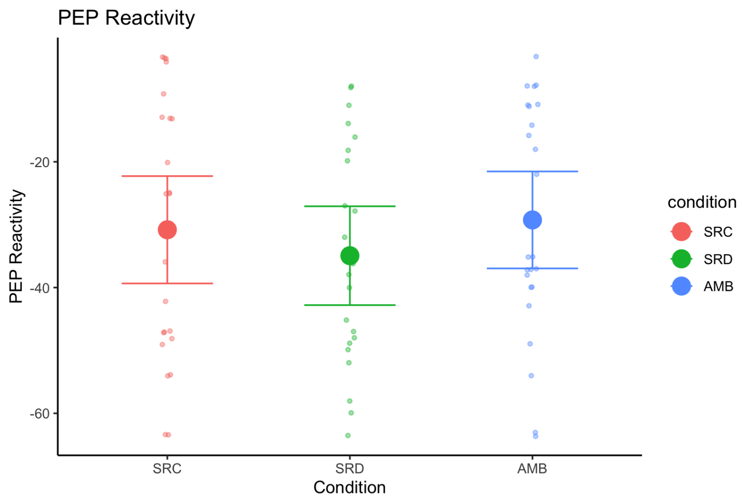
PEP Reactivity
PEP Reactivity
-20
-40
-60
SRC
SRD
AMB
Condition
condition
SRC
SRD
AMB

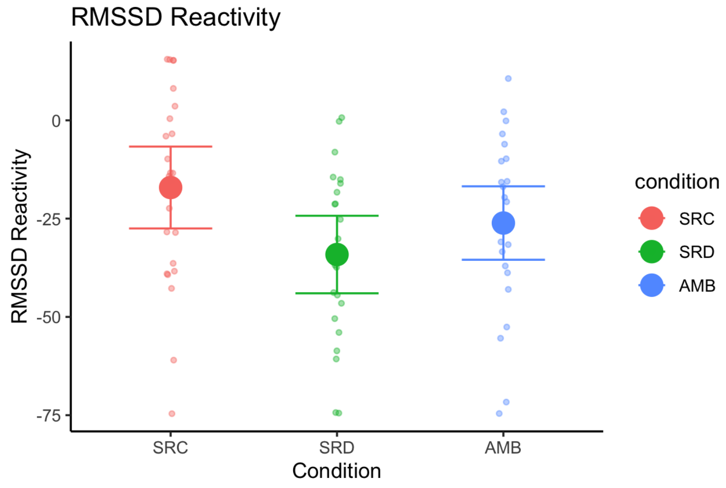
RMSSD Reactivity
RMSSD Reactivity
0
-25
-50
-75
SRC
SRD
AMB
Condition
condition
SRC
SRD
AMB

## Study 2

# Recruitment Flow Chart for Study 2

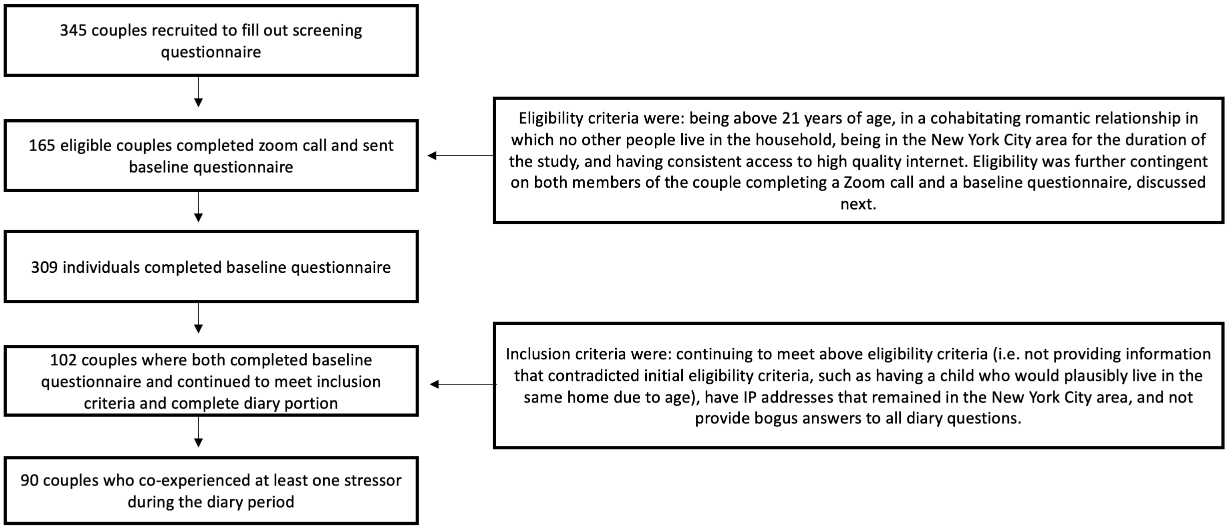

Supplement: Supplementary file 1 [file Data_Sheet_1.pdf]
